# A hypoxia related long non-coding RNA signature could accurately predict survival outcomes in patients with bladder cancer

**DOI:** 10.1080/21655979.2021.1948781

**Published:** 2021-07-19

**Authors:** Facai Zhang, Xiaoming Wang, Huan Hu, Yubo Yang, Jiahao Wang, Yin Tang, Dengxiong Li, Yunjin Bai, Ping Han

**Affiliations:** a Department of Urology/Institute of Urology, West China Hospital, Sichuan University, Sichuan Province, China; b Department of Urology, The Affiliated Hospital of Guizhou Medical University, Guizhou Province, China; c School of Clinical Medicine, Guizhou Medical University, Guizhou Province, China; d Department of Urology, The Second People’s Hospital of Yibin, Sichuan Province, China

**Keywords:** Bladder cancer, hypoxia, long noncoding rna, prognosis, TCGA

## Abstract

Hypoxia plays a significant role in tumor progression. This study aimed to develop a hypoxia-related long noncoding RNA (lncRNA) signature for predicting survival outcomes of patients with bladder cancer (BC). The transcriptome and clinicopathologic data were downloaded from The Cancer Genome Atlas (TCGA) database. Univariate Cox regression analysis and Lasso regression analysis were used to screened lncRNAs. Ten lncRNAs were screened out and included into the hypoxia lncRNA signature. The risk score based on hypoxia lncRNA signature could accurately predict the survival outcomes of BC patients. Immune infiltration analysis showed that six types of immune cells had significant different infiltration. Tumor mutation burden (TMB) analysis showed that the risk scores between the wild types and the mutation types of TP53, FGFR3, and RB1 were significantly different. Gene Set Enrichment Analysis (GSEA) showed that cancer-associated pathways belonged to the high risk groups and immune-related signal pathways were enriched into the low risk group. Then, we constructed a predictive model with the risk score, age, and clinical stage, which showed a robust prognostic performance. An lncRNA-mRNA coexpression network was constructed, which contained 62 lncRNA-mRNA links among 10 lncRNAs and 40 related mRNAs. In summary, the hypoxia lncRNA signature could accurately predict prognosis, chemotherapy and immunotherapy response in patients with BC and was relevant to clinicopathologic parameters and immune cell infiltration.

## Graphical Abstract

**Figure:**
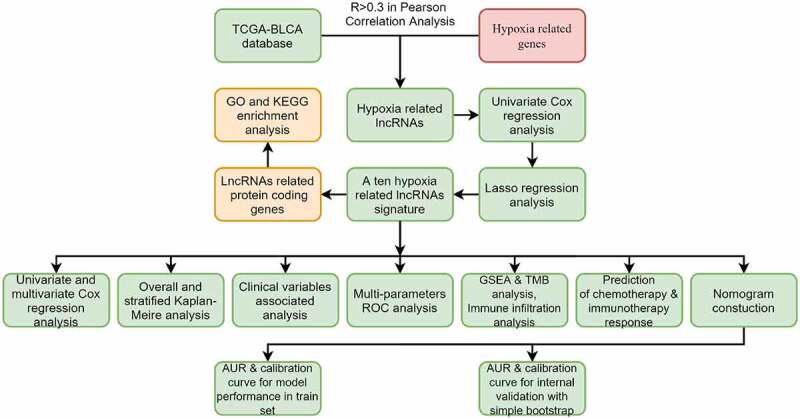

## Introduction

Bladder cancer (BC) is the second most common cancer in urinary and male reproductive system, and has a clear male predominance [1,2]. More than 90% of cases were urothelial carcinoma and 25% of patients with BC were presented with muscle invasive disease [3]. Although minimally invasive technology, neoadjuvant chemotherapy, and radiotherapy were introduced to treat BC, the survival rate of patients does not improve in the past 30 years [1,4]. Moreover, the staging system based on pathological parameters still requires improvement to predict patients’ survival accurately [5]. Recently, an increasing number of researches have focused on the molecular biomarkers [3]. The molecular subtype’s classes of BC could stratify responses to treatment and predict the survival outcomes effectively, which might be incorporated into clinical management in the future [6,7].

The rapid proliferation of tumor cells and the aberrant vascularization in tumor stroma resulted in hypoxia status in tumor microenvironment [8]. Tumor cells increased transcriptional activity of hypoxia inducible factor-1 (HIF-1) and hypoxia inducible factor-2 (HIF-2) so as to adapt hypoxia microenvironment [9]. Hypoxia-inducible factors could reprogram tumor cells and improve tumors’ malignancy, such as proliferation, invasion, metastasis, angiogenesis, immune invasion, and so forth [9–13]. Moreover, some researches have reported that hypoxia in tumor microenvironment had close relationships with treatment resistance, including radioresistance, chemoresistance, and immunosuppression [13–15]. Therefore, hypoxia-associated molecules biomarkers might be valuable to predict survival outcomes in patients with BC [16,17].

LncRNAs, a subtype of noncoding RNA family, have more than 200 nucleotides in their transcripts and play an indispensable roles in regulating initiation and progression of tumors [5,18]. To date, lots of studies have investigated hypoxia-associated lncRNAs and revealed their important regulatory roles in cancer cells [19–23]. It was reported that lncRNA-RMRP, lncRNA SNHG3, lncRNA GAS6-AS2, lncRNA CCAT1, and lncRNA GClnc1 could promote proliferation, migration, and invasion of BC cells [24–28]. Chen once reported that lncRNA LNMAT1 in cytoplasm promoted lymphatic invasion in BC. Meanwhile, lymphatic endothelial cell internalizing lncRNA LNMAT1 in exosomes could improve tube formation and migration *in vitro* [29,30]. It is due to the significance of lncRNA that Qian once conducted a meta-analysis to analyze the association between lncRNA expression and survival, and demonstrated that lncRNA could serve as diagnostic and prognostic biomarkers in BC.

Whether we could build a hypoxia-associated lncRNA signature to predict the prognosis of BC patients more accurately compared with traditional clinicopathologic parameters, and to provide some clinical guidance for chemotherapy and immunotherapy has become our goal of the study. Herein, we have successfully constructed a hypoxia-related lncRNA signature and investigated its performance and relationships with other clinicopathological variables in BC. Interestingly, the hypoxia related lncRNA signature could accurately predict the chemotherapy and immunotherapy response in patients with different risk scores, which may contribute to decision-making in the management of BC.

## Materials and methods

### Data collection

The transcriptome and clinical data of patients were downloaded from the TCGA database (https://portal.gdc.cancer.gov/). Patients with survival time less than 30 days were excluded. Finally, 396 cases were incorporated into our study. The Ensembl human genome browser (http://grch37.ensembl.org/index.html) was used to classify and annotate lncRNAs and protein-coding genes. Moreover, hallmark genes of hypoxia (total 200 genes) were downloaded from the hallmark gene sets of the Molecular Signatures Database (https://www.gsea-msigdb.org/gsea/msigdb/index.jsp). Since data of participants were acquired from the public database, written informed consent and approval of the ethics committee were waived.

### Identification of hypoxia associated LncRNAs

The ‘limma’ package was used in the R software to calculate Pearson correlation coefficients, which were employed to analyze the correlation between hypoxia hallmark genes and lncRNAs in TCGA dataset. The hypoxia-related lncRNAs with absolute value of correlation coefficient greater than 0.3 (|R|>0.3) and *P* value less than 0.05 (*P* < 0.05) were screened out and used to construct the hypoxia-related signature [5].

### Construction of the prognostic-related hypoxia LncRNA signature

All hypoxia-related lncRNAs were screened using univariate Cox regression analysis, to identify prognosis associated lncRNAs with *P*-value < 0.05. Then, the screened prognosis-associated lncRNAs were used to make Gene Ontology (GO) enrichment analysis and Kyoto Encyclopedia of Genes and Genomes (KEGG) pathway analysis with ‘clusterProfiler’ package and ‘enrichplot’ package in R software. Moreover, the screened prognosis-related hypoxia lncRNAs were incorporated into Lasso regression model [31], in which penalties were applied to all prognosis-associated hypoxia lncRNAs for preventing overfitting effects of the model. Penalty parameter (λ) for the model was determined by 10-fold cross-validation following the criteria that error was within 1 standard error of the minimum. After that, the selected lncRNAs constituted the hypoxia signature and could generate risk scores in multivariate Cox regression model with the following formula:

$$Riskscore=\overset{n}{\underset{i=1}{\sum }}coefficient_{i}*EXP{\left(mRNA\right)}_{i}$$

### Evaluation of the hypoxia LncRNA signature

All BC patients were divided into two groups in the light of the mean risk score, and the Kaplan–Meier method was used to compare overall survival (OS) of patients in different risk groups [32]. Principle component analysis (PCA) was used to evaluate the distribution of genes expression in patients with different risk levels. Stratified survival analysis was performed to examine the accuracy and stability of the hypoxia lncRNAs signature. Risk scores were compared in subgroups stratified by clinicopathological parameters to explore potential relationships between the signature and clinicopathological parameters.

### Immune infiltration analysis, tumor mutational burden analysis, and gene set enrichment analysis

The CIBERSORT tool was used to estimate the contents of 22 human immune cells in each BC patient. Next, we compared the infiltrating immune cells in the high- and the low-risk groups and identified the significantly different immune infiltrating cells [11].

The tumor mutational data were downloaded from TCGA and the maftools package were used to analyze the mutational data in both the high- and the low-risk groups. TMB was calculated with the tumor specific mutation genes [33]. We listed the top mutational genes and compared the risk scores in mutational- and wild-type cohorts.

We uploaded RNA-seq profiles to GSEA to investigate that differentially expressed gene-related signaling pathways in the high-risk group and the low-risk group. The enriched set were screened based on a FDR < 0.25 and *P* < 0.05 after 1000 permutations [32].

### Correlation of the hypoxia signature with clinical parameters

The risk score and other clinical parameters in the TCGA dataset were incorporated into univariate Cox regression and multivariate Cox regression to evaluate whether the risk score was an independent prognostic predictor, and then ROC curves were used to calculate the predictive accuracy of the risk score and other clinicopathological parameters [33].

### Development of a predictive nomogram based on clinical parameters and the risk score

Age, gender, AJCC-stage, grade, and hypoxia-related risk score were incorporated into the univariate Cox regression analysis and multivariate Cox regression analysis, and then ROC curves were used to evaluate the predictive accuracy of the risk score and other clinicopathological parameters. After that, we picked up the independent predictive factors with *P* < 0.05 and AJCC-stage to build a Cox regression model and presented it with a nomogram to facilitate clinical practice [33]. The area under curve (AUC), Brier scores, and calibration plots were used to assess the discrimination and calibration of the model in 1, 3, and 5 years. The simple bootstrap strategy was used to validate the model internally [32,33].

### Construction of the LncRNA-mRNA coexpression network and function enrichment analysis

Pearson correlation analysis was used to explore hypoxia lncRNAs correlated mRNAs with absolute value of correlation coefficient greater than 0.3 (|R| > 0.3) and *P* value less than 0.05 (*P* < 0.05) [5]. Next, the Cytoscape software (Version 3.7.2) (https://cytoscape.org/) was applied to construct and visualize the lncRNA-mRNA coexpression network. The hypoxia lncRNA-associated mRNAs were uploaded to DAVID database (https://david.ncifcrf.gov/home.jsp) to make GO enrichment analysis and KEGG pathway analysis. *P* < 0.05 was deemed as statistically significant [5].

### Prediction of chemotherapy response

Public pharmacogenomics database Genomics of Drug Sensitivity in Cancer (GDSC) was used to evaluate and predict the chemotherapy response of BC patients in different risk groups in TCGA database [34]. The half-maximal inhibitory concentration (IC50), a significant predictor of chemosensitivity, was compared between the high and low risk groups, and *P* < 0.05 was considered statistically significant.

### Prediction of immunotherapy response

TMB, Programmed cell death 1 ligand 1 and 2 (PD-L1 and PD-L2), and microsatellite instable (MSI) in tumor tissue were deemed as potent biomarkers for predicting immunotherapy response [35]. Therefore, we first analyzed the TMB in both risk groups and explored the correlation between risk score and TMB. Then, the expression of CD274 (PD-L1) and PDCD1LG2 (PD-L2) in both different risk groups were calculated and compared at the transcriptional level. Moreover, we also calculated and compared the transcriptional expression of significant mismatch repair genes in tumor samples, including MLH1, MSH2, MSH6, and PMS2. Finally, we used The Cancer Immunome Atlas (TCIA) database (https://tcia.at/) to generate the immunophenoscore (IPS) in each sample, which was a superior predictor of response to anti-cytotoxic T lymphocyte antigen-4 (CTLA-4) and anti-programmed cell death protein 1 (PD-1) [36], and then the IPS in different risk groups were compared to explore the relationships between risk scores and IPS. *P* < 0.05 was consideredstatistically significant.

## Statistical analysis

All statistical analyses were performed by using the R software (Version 4.0.2) (https://www.r-project.org/) and GraphPad Prism 8. Quantitative data in two groups were compared using Student *t*-test and quantitative data in three or more groups were compared with one-way analysis of variance (ANOVA) or Welch’s test. *P* < 0.05 was considered statistically significant.

## Results

BC is a common cancer in urinary and male reproductive system. The rapid proliferation of tumor cells and the aberrant vascularization in tumor stroma resulted in hypoxia status in tumor microenvironment, which could enhance the invasion of malignancies and had closely relationships with treatment resistance. Along with the wide application of next-generation sequence (NGS) in clinical practice, an increasing number of biomarker-oriented studies have become more and more popular. Herein, we aimed to build a hypoxia-associated lncRNA signature to predict the prognosis of BC patients more accurately compared with traditional clinicopathologic parameters, and to be helpful for decision-making, especially for those patients required chemotherapy and immunotherapy.

### Selection of hypoxia-related LncRNA and construction of hypoxia LncRNA signature

**Figure 1.**
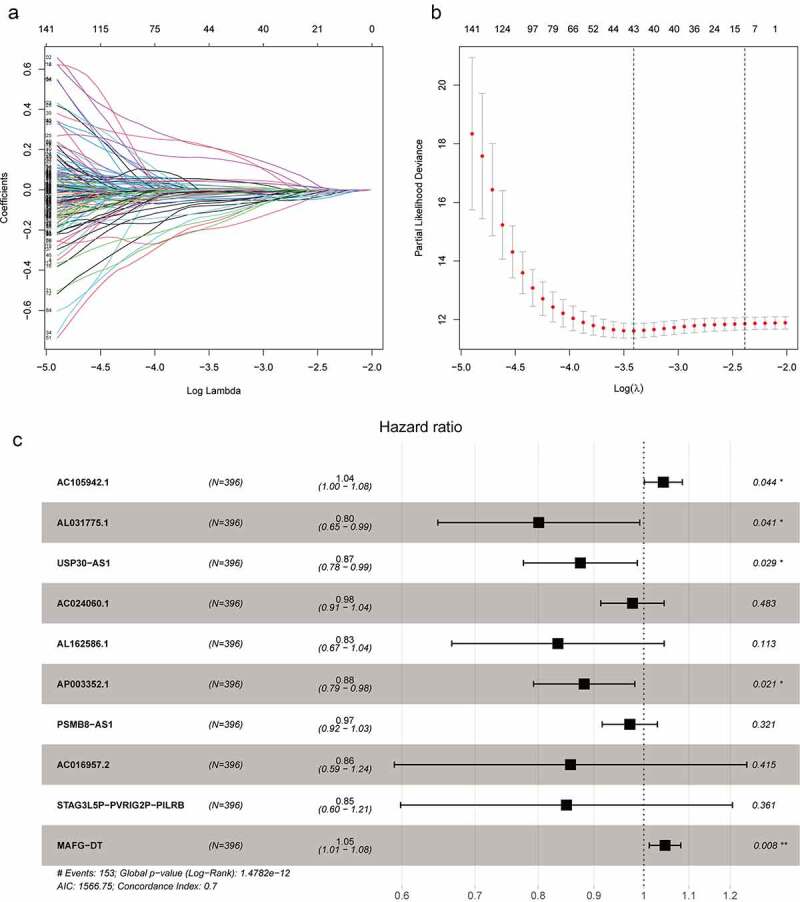
The selection of hypoxia-related lncRNA utilizing Lasso regression analysis. (a, b) The prognosis-related hypoxia lncRNAs screened by univariate Cox regression were incorporated into Lasso regression analysis. Penalty parameter (λ) for the model was determined by 10-fold cross-validation following the criteria that error is within 1 standard error of the minimum. (c) Ten prognosis-related hypoxia genes were incorporated into multivariate regression model and used to generate hypoxia-associated risk scores

The flowchart showed the main procedures of our study (Graphical Abstract). The basic characteristics of BC patients in the TCGA database were presented in Table 1. We classified the RNA sequencing data and extracted 14,142 lncRNAs in TCGA database. Meanwhile, a total of 200 hypoxia-related genes were acquired from Molecular Signature Database, among which 195 genes were expressed in BC samples (Supplementary Table 1). Pearson correlation analysis were applied to analyze the correlation between hypoxia related genes and lncRNAs, and 1423 hypoxia related lncRNAs were identified with the criteria that |R| > 0.3 and *P* < 0.05 (Supplementary Table 2). We first analyzed 1423 hypoxia-associated lncRNAs with GO enrichment and found that these lncRNAs were relevant to catabolic or metabolic process, which were in accordance with hypoxia-related biological process (Supplementary Figure 1). Then, KEGG enrichment was applied to explore the associated enriched pathways, but no significant pathways were enriched due to the lack of noncoding RNA information in enriched pathways. Univariate Cox regression analysis were used to select prognosis related lncRNAs among the 1423 hypoxia-related lncRNAs and 248 prognosis-related hypoxia lncRNAs were screened out (Supplementary Table 3). In order to further screen variables and prevent overfitting, lasso regression analysis was used and finally 10 prognosis-related hypoxia lncRNAs were selected to construct the hypoxia lncRNA signature (Figures 1a and 1b). Among them, eight lncRNAs (AL031775.1, USP30-AS1, AC024060.1, AL162586.1, AP003352.1, PSMB8-AS1, AC016957.2, and STAG3L5P-PVRIG2P-PILRB) were deemed as protective predictors with hazard ratio < 1. While AC105942.1 and MAFG-DT were regard as harmful with hazard ratio > 1 (Figure 1c).

**Table 1.** Baseline Clinical Characteristics of TCGA Database

Variables	Number	Risk score		P
		Mean	Standard deviation	
**Total**	396	1.324	1.048	
**Age**				0.167
<60	86	1.124	0.719	
60–70	126	1.317	0.951	
70–80	129	1.359	0.949	
≥80	55	1.439	0.963	
**Gender**				0.467
Female	104	1.362	0.892	
Male	292	1.286	0.916	
**Grade**	3 cases missing			0.019
Low grade	18	0.855	0.497	
High grade	375	1.331	0.921	
**AJCC-stage**	2 cases missing			<0.001
I	2	0.485	0.141	
II	124	1.037	0.650	
III	138	1.349	0.913	
IV	130	1.527	1.046	
**T**	33 cases missing			<0.001
T1	3	0.578	0.190	
T2	113	1.066	0.669	
T3	190	1.418	0.966	
T4	57	1.479	1.057	
**N**	41 cases missing			0.037
N0	229	1.211	0.825	
N1	44	1.529	1.170	
N2	75	1.480	0.989	
N3	7	1.339	0.778	
**M**	197 cases missing			0.011
M0	189	1.180	0.855	
M1	10	1.903	1.081

### Evaluation of the hypoxia LncRNA signature

**Figure 2.**
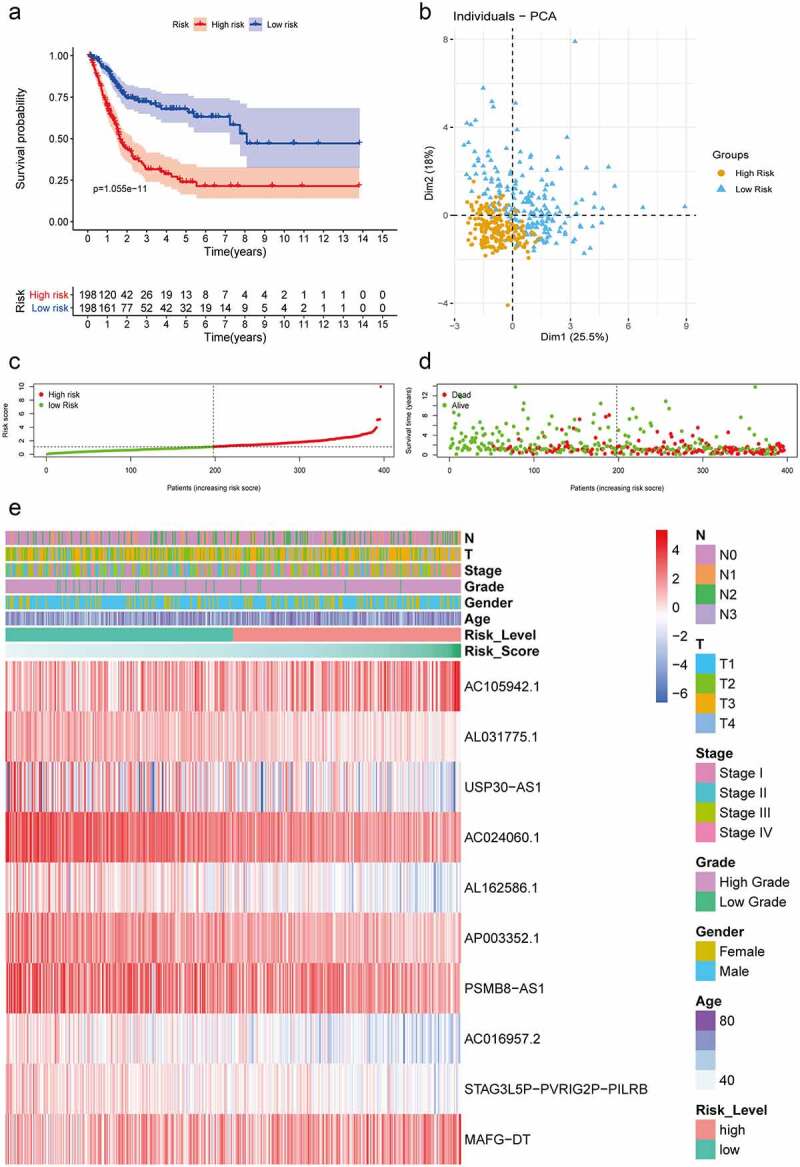
Construction and validation of hypoxia-associated prognostic lncRNA signature. (a) Kaplan–Meier analysis showed that the overall survival of patients in the low-risk groups were longer than those in the high-risk groups in the TCGA database. (b) Principal components analysis based on the hypoxia lncRNAs signature demonstrated that two distinctly different distribution patterns between high-risk and low-risk groups. (c) Distribution of risk scores of patients with different risk scores based on the hypoxia-related lncRNA prognostic signature (d) Scatter plot showed that patients with lower risk scores had better survival and lower mortality risk. (e) Multigroup heatmap of the hypoxia lncRNA signature showed that AC105942.1 and MAFG-DT increased notably with the increment of risk scores, while the expressions of AL031775.1, USP30-AS1, AC024060.1, AL162586.1, AP003352.1, PSMB8-AS1, AC016957.2, and STAG3L5P-PVRIG2P-PILRB decreased distinctly

All 10 prognosis-related hypoxia lncRNAs were incorporated into multivariate Cox regression analysis to generate risk scores in the light of the formula described in Method and Materials. The patients with different risk scores were divided into high-risk and low-risk groups using the mean risk score as a cutoff value in the TCGA database. Kaplan–Meier analysis showed that patients in the low-risk group had better OS than that in the high-risk group, which demonstrated the excellent discrimination of this signature (Figure 2a). Principle component analysis based on gene expression showed two significantly distinct distribution between patients in high- and low-risk groups (Figure 2b). As for patients with lower risk scores, they usually had lower mortality rates and longer survival time compared with ones with higher risk scores (Figures 2c and 2d). Furthermore, the expressions of AC105942.1 and MAFG-DT increased notably with the increment of risk scores, while the expressions of AL031775.1, USP30-AS1, AC024060.1, AL162586.1, AP003352.1, PSMB8-AS1, AC016957.2, and STAG3L5P-PVRIG2P-PILRB decreased distinctly, which were in accordance with hazard ratio of these predictors (Figure 2e).

### Relationship between hypoxia LncRNA signature and clinicopathological parameters

**Figure 3.**
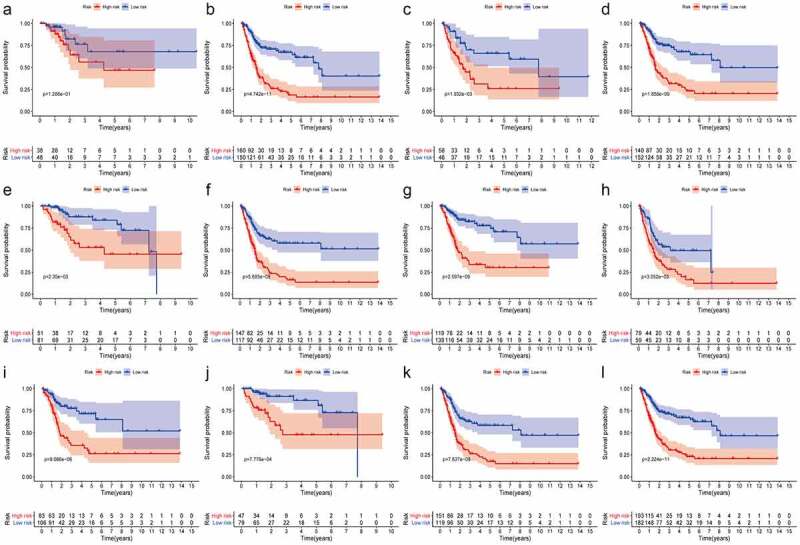
The Kaplan–Meier analysis showed that bladder cancer patients with lower risk scores still had better OS than the ones with higher risk scores in subgroups of age ≥ 60 (b), female (c), male (d), low T-stage (e), high T-stage (f), nodal metastasis-free (g), nodal metastasis (h), metastasis-free subgroup (i), high AJCC-stage (j), low AJCC-stage (k), and high pathological grade (l). While the difference of OS was not significant in the subgroups of age ≤ 60 (a)

First, in order to further verify accuracy of this hypoxia lncRNA signature, patients in the TCGA database were stratified according to age (≥ 60 y and < 60 y), gender (female and male), AJCC stage (I+ II and III+IV), T stage (T1-T2 and T3-T4), N stage (N0 and N1-3), M stage (M0 and M1), and pathological grade (low and high), and the Kaplan–Meier analysis showed that patients with low risk scores had better OS compared with ones with high-risk scores in the subgroups of age ≥ 60 (*P* = 4.742e−11), female (*P* = 1.932e−03), male (*P* = 1.858e−09), low T-stage (*P* = 2.35e−03), high T-stage (*p* = 5.685e−08), nodal metastasis-free (*p* = 2.597e−09), nodal metastasis (*P* = 3.052e−03), metastasis-free subgroup (*P* = 9.086e−06), high AJCC-stage (*P* = 7.127e−03), low AJCC-stage *(P *= 7.776e−04), and high pathological grade (*P* = 2.224e−11) (Figure 3b-l), while the difference of OS time was not significant in subgroups of age ≤ 60 (*P* = 1.286e−01), metastasis subgroup (*P* = 2.802e−01), and low pathological grade (*P* = 1e+00) (Figure 3a and Supplementary Figure 2).

**Figure 4.**
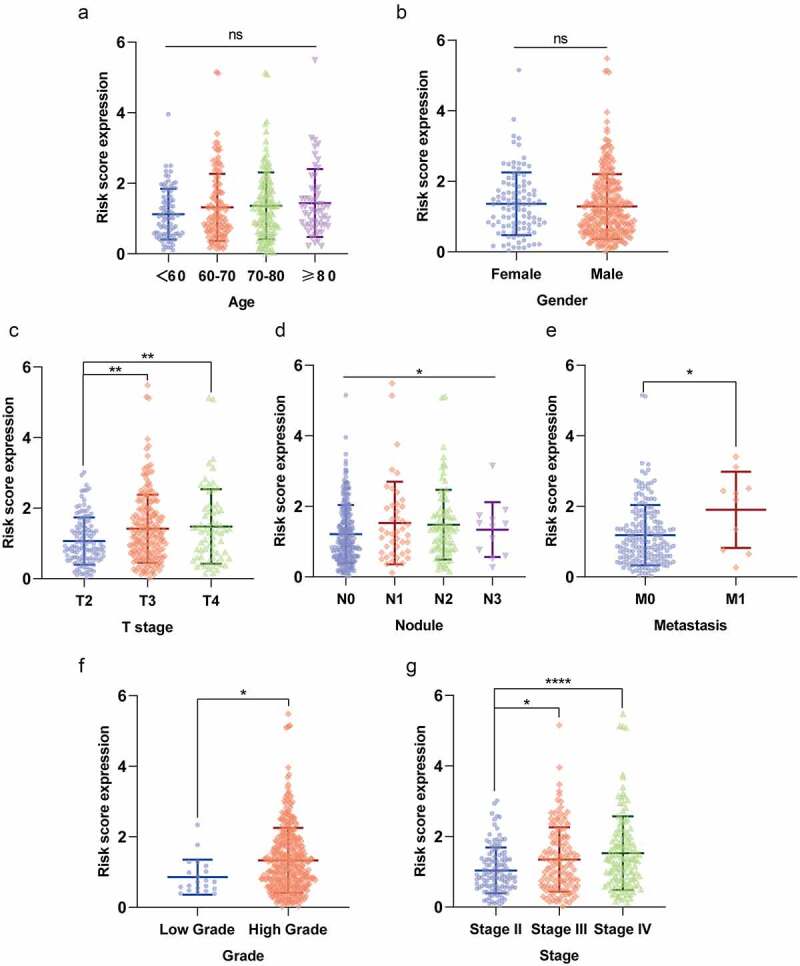
The risk scores of patients in different subgroups stratified by clinicopathologocal parameters. The risk scores were significantly different in subgroups of (c) advanced T stage (T3 and T4), (d) nodule-metastasis (N1-N3), (e) metastasis (M1), (f) high pathological grade, and (g) advanced AJCC stage (Stage III and Stage IV). Moreover, the difference of the risk score in different age subgroups and gender subgroups was not significant (a and b). *P < 0.05; **P < 0.01; *** *P* < 0.001; **** *P* < 0.0001; ns: not significant

Secondly, we compared the risk scores in different subgroups stratified by clinicopathological parameters and found that the risk scores were significantly higher in subgroups of advanced T stage (T3 and T4), nodule-metastasis (N1–N3), metastasis (M1), high pathological grade, and advanced AJCC stage (Stage III and Stage IV) (Figure 4c-g). It suggested that the risk score based on the hypoxia lncRNA signature had a correlation with pathologic parameters. Moreover, we observed that the difference of the risk score in different age subgroups and gender subgroups were not significant (Figures 4a and 4b).

### Tumor mutational burden analysis

**Figure 5.**
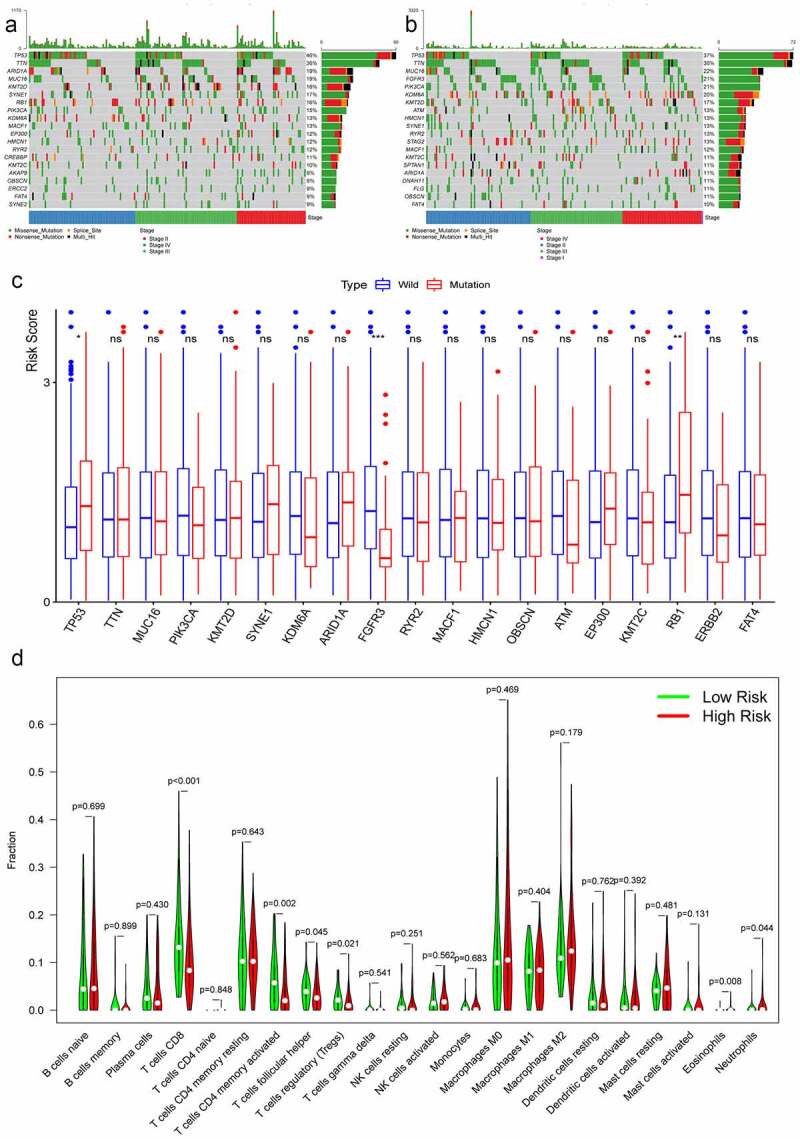
Tumor mutational burden analysis and immune cells infiltration analysis. The top 20 mutational genes were listed in high-risk group (a) and low-risk group (b). The risk scores between the wild type and the mutation type of the top frequent mutational genes were compared (c). The vioplots showed that 22 immune cells content in the high-risk and low-risk groups (d). *P < 0.05; **P < 0.01; *** *P* < 0.001; ns: not significant

The maftool package in R software was employed to summarize and analyze the mutational data in the TCGA database. In order to compare different mutational genes, the top 20 mutational genes were listed in high- and low-risk groups (Figures 5a and 5b). We found that TP53, TTN, ARID1A, MUC16, KMT2D, SYNE1, PIK3CA, KDM6A, MACF1, HMCN1, RYR2, KMT2C, OBSCN, and FAT4 were the most frequent mutational genes in both risk groups. RB1, EP300, CREBBP, AKAP9, ERCC2, and SYNE2 belonged to top 20 frequent mutational genes in the high-risk group, while FGFR3, ATM, STAG2, SPTAN1, DNAH11, and FLG were parts of the top 20 frequent mutational genes in the low-risk group. Furthermore, the risk scores between the wild type and the mutation type of the top frequent mutational genes were compared. The risk scores in the mutation types of TP53 and RB1 were significantly higher than that in the wild types, while the risk scores in the wild type of EGFR3 were higher than that in the mutational type (Figure 5c).

### Relationship between hypoxia signature and immune cells infiltration

Twenty-two immune cells types were evaluated in the TCGA database, and six sorts of immune cells types were significantly different between the high-risk group and the low-risk group, including CD8^+^ T cells, activated memory CD4^+^ T cells, follicular helper T cells, regulatory T cells, eosinophils, and neutrophils (Figure 5d). The contents of CD8^+^ T cells, activated memory CD4^+^ T cells, follicular helper T cells, and regulatory T cells were significantly higher in the low-risk group, while the contents of eosinophils and neutrophils were much higher in the high-risk group.

### Gene set enrichment analysis

**Figure 6.**
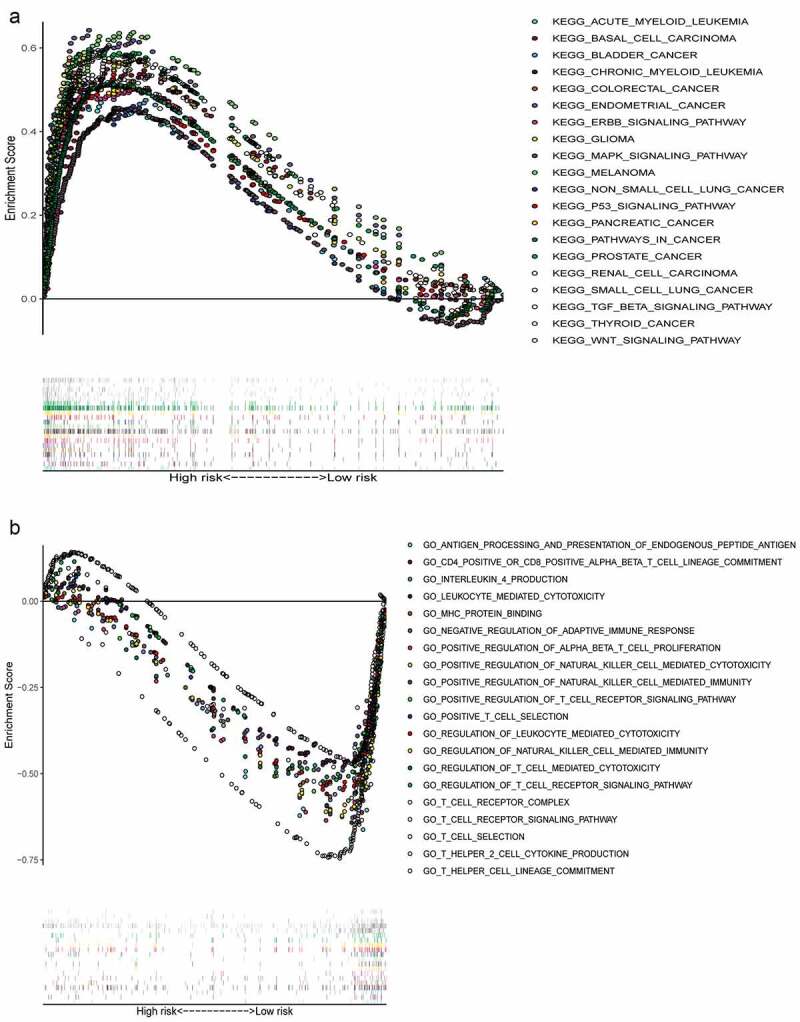
Multiple GSEA pathways in the high- and low-risk groups. GSEA results showed that significant enrichment of cancer-related signaling pathways in the high-risk group (a), and significant enrichment of immune-related signaling pathways in the low-risk group (b)

We collected the top 50 KEGG molecular pathways that differentially expressed genes enriched to in high-risk groups. Interestingly, some cancer-associated pathways were enriched into the high risk groups, including acute myeloid leukemia, basal cell carcinoma, BC, chronic myeloid leukemia, colorectal cancer, endometrial cancer, ERBB signaling pathway, MAPK signaling pathway, glioma, melanoma, nonsmall cell lung cancer, P53 signaling pathway, pancreatic cancer, pathways in cancer, prostate cancer, renal cell carcinoma, small cell lung cancer, TGF-β signaling pathway, thyroid cancer, and WNT signaling pathway (Figure 6a). Moreover, there were only six irrelevant KEGG pathways with FDR < 0.25 and *P* < 0.05 enriched into the low-risk group, so we made a Gene Ontology (GO) enrichment analysis and collected the top 50 GO molecular pathways in low risk group. Some immune-related signal pathways were enriched into the low-risk group, which suggested that activation of immune regulation and response related pathways might contribute to better OS (Figure 6b).

### The hypoxia related LncRNA signature is an independent prognostic factor

Age, gender, clinical stage, T stage, N stage, and the risk score were all incorporated into univariate Cox regression and multivariate Cox regression to screen out the independent prognostic factors. Due to the lack of metastatic data in more than half of patients, we ignored metastasis as an independent prognostic factor to minimize data loss in the TCGA database.

**Figure 7.**
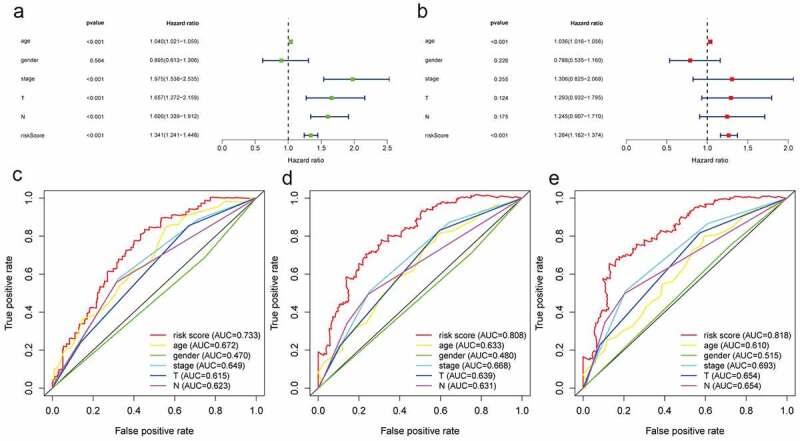
The predictive performance of the risk score and traditional clinicopathological parameters. (a and b) The univariate and multivariate Cox regression suggested that the risk score was an independent prognostic factor. The multiple ROC curves of the risk score and other clinicopathological parameters in 1, 3, and 5 years (c, d, e) demonstrated the excellent discrimination of the risk score based on hypoxia lncRNA signature

Age, clinical stage, T stage, N stage, and the risk score based on hypoxia lncRNA signature were all prognostic-associated factors with *P* < 0.05 in univariate Cox regression (Figure 7a). Multivariate Cox regression analysis showed that age and the hypoxia lncRNA-related risk score were independent prognostic factors (Figure 7b). The multivariate ROC showed that AUC of the risk score, age, gender, clinical stage, T stage, and N stage were 0.733, 0.672, 0.470, 0.649, 0.615, and 0.623 in 1 year time; 0.808, 0.633, 0.480, 0.668, 0.639, and 0.631 in 3 year time; 0.818, 0.610, 0.515, 0.693, 0.654, and 0.654 in 5 year time (Figure 7c-e). It suggested that compared with traditional pathological prognostic factors, the hypoxia lncRNA-related risk scores had a preeminent prognostic performance.

### Prognostic nomogram construction

**Figure 8.**
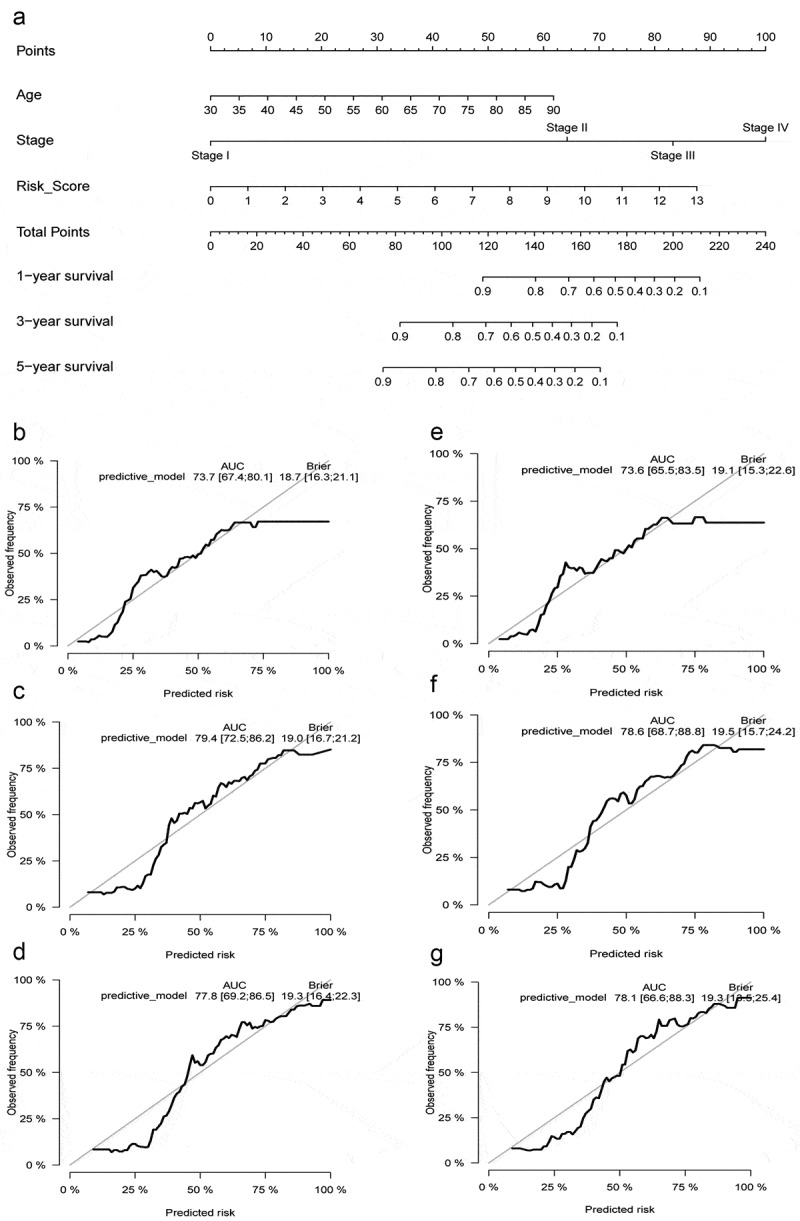
Construction and internal validation of the nomogram with the risk scores based on hypoxia lncRNA signature and other traditional clinicopathological parameters. The predictive model was presented with a nomogram (a), and the predictive performance of the nomogram in 1, 3, and 5 years in the training set (b, c, d), internal validation set (e, f, g)

We constructed a prognostic model presented with a nomogram, including the independent prognostic factors (age and the risk score) and traditional clinical stage in multivariable Cox regression analysis (Figure 8a). The Concordance index was 0.722 for the predictive model. AUC and Brier scores of the prognostic model in 1, 3, and 5 years were 73.7 [67.4;80.1] and 18.7 [16.3;21.1], 79.4 [72.5;86.2] and 19.0 [16.7;21.2], 77.8 [69.2;86.5] and 19.3 [16.4;22.3] in the whole TCGA database (training set), respectively (Figure 8b–d). Similarly, we used 1000 times bootstrap strategy to validate the model internally and the AUC and Brier scores in 1, 3, and 5 years were 73.6 [65.5;83.5] and 19.1 [15.3;22.6], 78.6 [68.7;88.8] and 19.5 [15.7;24.2], 78.1 [66.6;88.3] and 19.3 [13.5;25.4] in internal validation set (Figure 8e–g).

### Construction of the LncRNA–mRNA coexpression network and functional enrichment analysis

**Figure 9.**
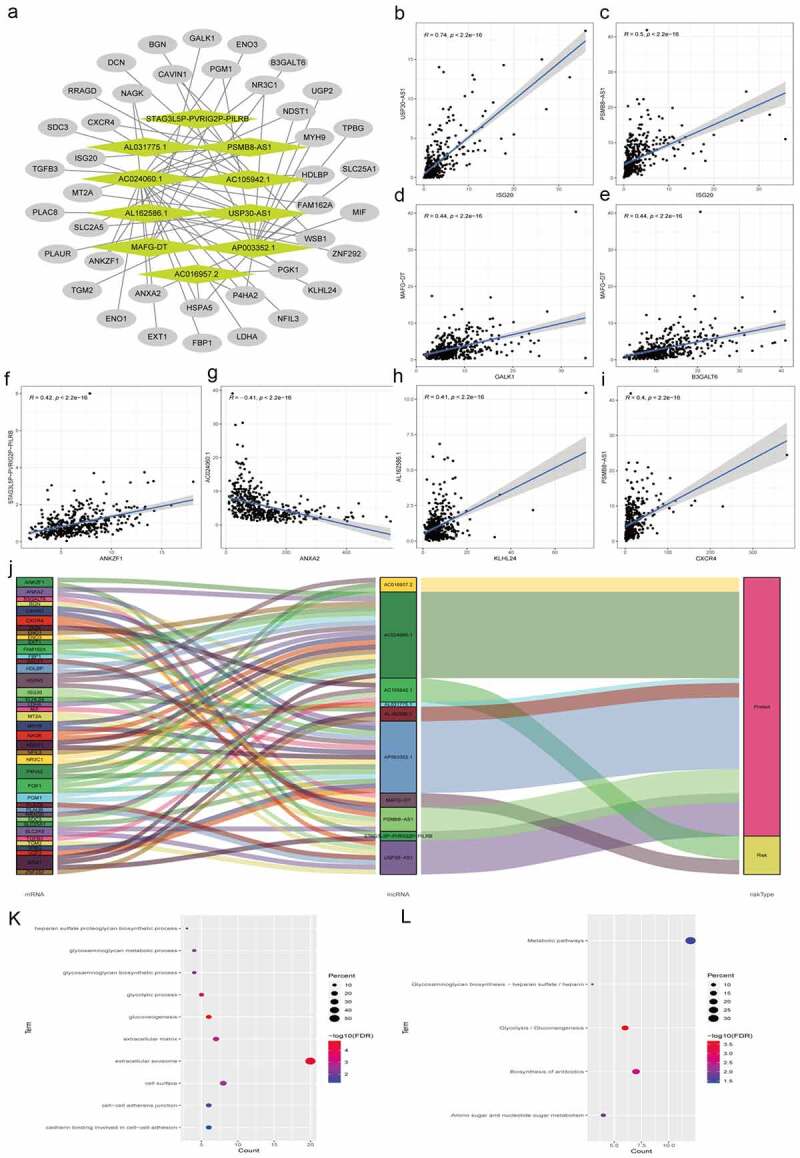
Construction of the hypoxia-related lncRNA–mRNA coexpression network and functional enrichment analyses. (a) The lncRNA-mRNA co-expression network showed that 62 lncRNA-mRNA links among 10 lncRNAs and 40 related mRNAs. (b-i) 8 lncRNA-mRNA links with correlation coefficient larger than 0.4 were presented. Except the negative correlation between AC024060.1 and ANXA2, all 7 lncRNA-mRNA links were positive correlation. (j) The Sankey diagram shows the connection between the 40 mRNAs and 10 hypoxia-related lncRNAs and also illustrated their protective or risk properties. (k) Gene Ontology (GO) analysis showed the top 10 terms associated with the mRNAs that coexpressed with the 10 hypoxia-related lncRNAs. (l) Kyoto Encyclopedia of Genes and Genomes (KEGG) pathway analysis showed the top 10 enriched signaling pathways associated with the mRNAs that coexpressed with the 10 hypoxia-related lncRNAs

We used Pearson correlation analysis to screen out 40 hypoxia lncRNA-related mRNAs with |R|>0.3 and *P* < 0.05. An lncRNA-mRNA coexpression network were constructed, which contained 62 lncRNA-mRNA links among 10 lncRNAs and 40 related mRNAs (Figure 9a and Supplementary Table 4). Then, we summarized and presented eight lncRNA-mRNA links with correlation coefficient larger than 0.4 (Figure 9b–i). As shown in the Figure 9, all seven lncRNA-mRNA links were positive correlation except AC024060.1 negatively correlated with ANXA2. The Sankey plot showed the relationships between 40 mRNAs and 10 hypoxia lncRNAs and clearly classified lncRNAs in the light of their biological functions (protect or risk) (Figure 9j). Top 10 GO enrichment terms were presented in Figure 9k and KEGG pathway analysis showed that metabolism and biosynthesis pathways were enriched, which were in concordance with hypoxia and reprogramming (Figure 9l).

### Prediction of chemotherapy response

**Figure 10.**
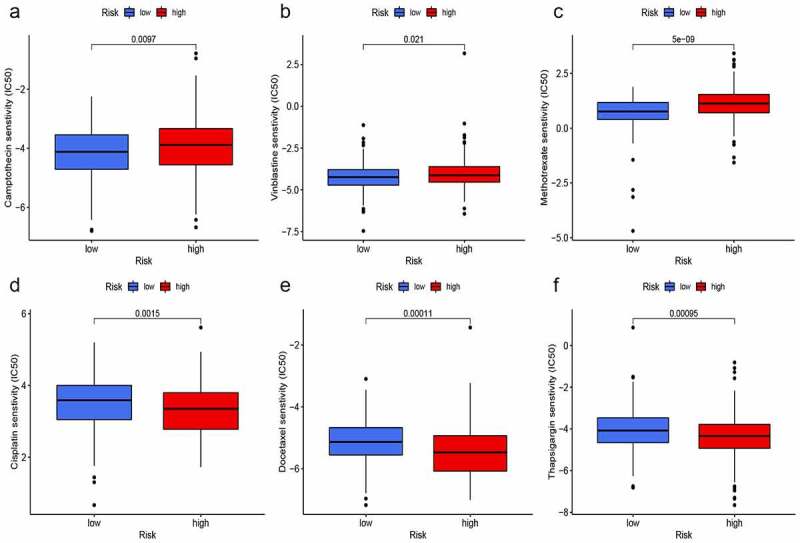
**Prediction of chemotherapy response**. IC50 of camptothecin (a), vinblastine (b), and methotrexate (c) in the high-risk group were significantly higher than that in the low-risk group. On the contrary, IC50 of cisplatin (d), docetaxel (e), and thapsigargin (f) were lower in the high-risk group

We used the ‘pRRophetic’ package to explore the GDSC database, and to investigate whether the risk score based on hypoxia lncRNAs could predict chemotherapy response in different risk groups. Gemcitabine (G), cisplatin (C), methotrexate (M), vinblastine (V), and doxorubicin (A) were selected to evaluate chemotherapy response in both risk groups, for they constituted fundamental GC or MVAC protocol in muscle-invasive BC [32]. Moreover, camptothecin, docetaxel, and thapsigargin were also used to evaluate response, for thapsigargin could induce endoplasmic reticulum stress-associated apoptosis in BC and camptothecin and docetaxel were widely used for intravesical chemotherapy in nonmuscle-invasive BC [37]. Interestingly, IC50 of camptothecin (Figure 10A), vinblastine (Figure 10B), and methotrexate (Figure 10C) in the high-risk group were significantly higher than that in the low-risk group, which meant that the chemotherapy response rates of these drugs were lower in the high-risk group. On the contrary, IC50 of cisplatin (Figure 10D), docetaxel (Figure 10E), and thapsigargin (Figure 10F) were lower in the high-risk group, which indicated that the application of these drugs could be more beneficial for patients with higher-risk scores. Furthermore, the differences of IC50 in Gemcitabine and doxorubicin in both groups were not significant.

### Prediction of immunotherapy response

**Figure 11.**
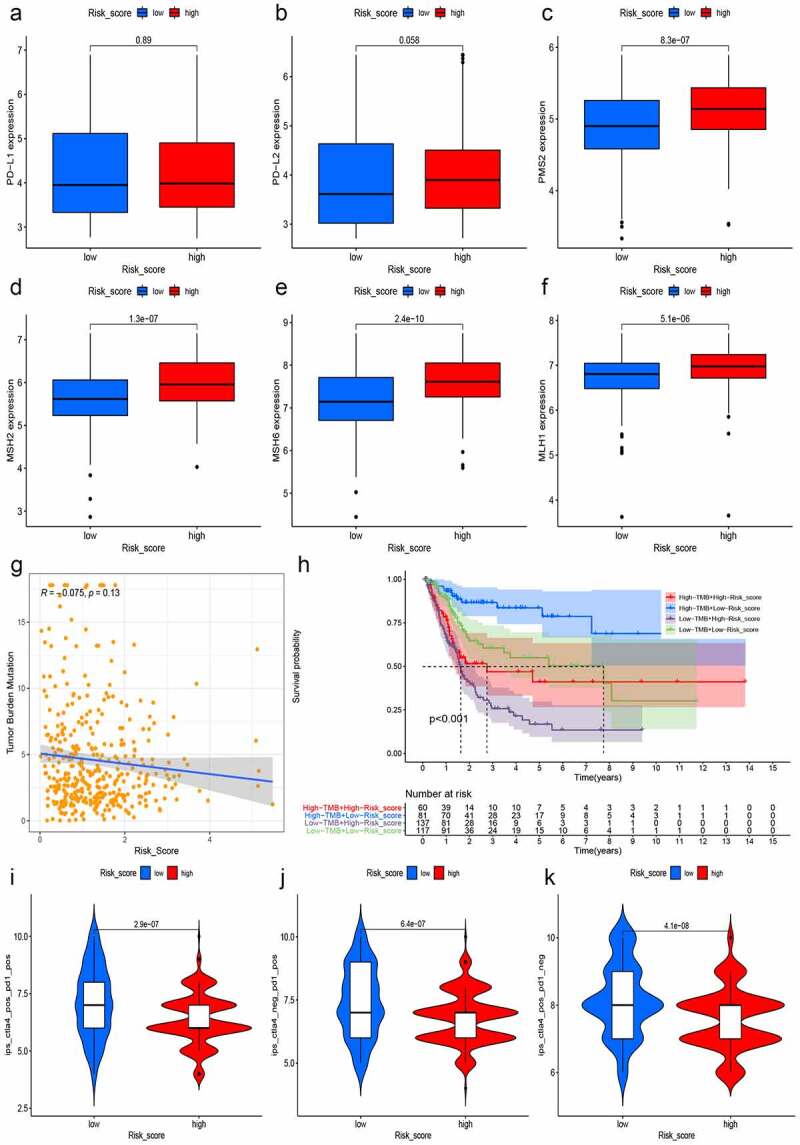
**Prediction of immunotherapy response**. The expressions of PD-L1 and PD-L2 were slightly higher in the high-risk group with *P* > 0.05 (a and b). The expression of mismatch repair genes in tumor samples, MLH1 (c), MSH2 (d), MSH6 (e), and PMS2 (f), expressed significantly higher in the high-risk group. Moreover, with the increment of risk scores, TMB decreased slightly with R = −0.075 and P = 0.13 (g) and the Kaplan–Meier curves showed that patients with higher TMB and low-risk scores tended to have the best overall survival and those with low TMB and high risk scores usually had worst survival probabilities (h). The IPS of anti-CTLA-4 (i), anti-PD-1(j), and anti-(CTLA-4 plus PD-1) (k) in the high-risk group was significant lower than that in the low-risk group, predicting that patients with higher risk scores had a worse immunotherapy response

TMB, PD-L1/PD-L2, and MSI in tumor tissue were deemed as potent biomarkers for predicting immunotherapy response [33,35]. We first analyzed the expression of CD274 (PD-L1) and PDCD1LG2 (PD-L2) in both different risk groups and found that PD-L1 and PD-L2 were slightly higher in the high-risk group, but the difference was not statistically significant (Figures 11A and 11b). Then, we calculated and compared the transcriptional expression of significant mismatch repair genes in tumor samples and found that all four mismatch repair genes (MLH1, MSH2, MSH6, and PMS2) expressed significantly higher in the high-risk group (Figure 11C–F), which signified that the microsatellites might be more stable in the high-risk group. Moreover, we also explored the relationship between the risk score and TMB, for an increasing number of studies showed that higher TMB predicted a better immunotherapy response [33]. Along with the increment of risk scores, the TMB decreased slightly with R = −0.075 and P = 0.13 (Figure 11G). The Kaplan–Meier curves showed that patients with higher TMB and low-risk scores tended to have the best OS and those with lower TMB and high-risk scores usually had worst survival probabilities (Figure 11H). Finally, we used the TCIA database to generate the IPS in each sample, which was a superior predictor of response to anti-CTLA-4 and anti-PD-1, and then the IPS of anti-CTLA-4 (Figure 11I), anti-PD-1 (Figure 11J), and anti-(CTLA-4 plus PD-1) (Figure 11K) in the high-risk group was significant lower than that in the low-risk group, which powerfully predicted that patients with higher risk scores had a worse immunotherapy response. Taken together, the all biomarkers above predicted that patients with higher risk scores tended to have worse immunotherapy response.

## Discussion

BC ranks the 11th among all diagnosed cancers in the world, which caused more than 16,000 deaths in the United State and have a clear male predominance [2,3,38]. While the 5-year survival has improved for many other malignancies, such as melanoma and hepatocellular carcinoma, it has remained largely unchanged in BC [1]. To date, an increasing number of researches have focused on hypoxia, a hallmark of tumor microenvironment, for it is closely related to reprogramming, invasion, and metastasis of tumor [8–10]. Hypoxia-related lncRNAs have been studied to seek out potential therapeutic targets. It was reported that lncRNA-RMRP, lncRNA SNHG3, lncRNA GAS6-AS2, lncRNA CCAT1, and lncRNA GClnc1 could promote proliferation, migration, and invasion in BC [24–28].

It is due to the significant roles of hypoxia-related lncRNAs that we first constructed a signature with hypoxia lncRNA to predict patients’ survival outcomes in BC. We used AC105942.1, AL031775.1, USP30-AS1, AC024060.1, AL162586.1, AP003352.1, PSMB8-AS1, AC016957.2, STAG3L5P-PVRIG2P-PILRB, and MAFG-DT to construct a hypoxia signature with a robust performance. The AUC of our signature in 1, 3, and 5 years were 0.733, 0.808, and 0.818, respectively, which were much higher than the AUC of AJCC-stage, T stage, and N stage (Figure 7). Then, we constructed a predictive model with risk score, age, and stage in TCGA database and presented it with a nomogram. 1000 times simple bootstraps were used to validate the nomogram internally. The Concordance index and AUC were used to evaluate the model’s discrimination, and calibration plot and Brier scores were applied to estimate the calibration of this model, which all demonstrated the model’s robust predict performance (Figure 8).

Next, we studied each lncRNA in our signature and found that AC024060.1, AC105942.1, AL031775.1, USP30-AS1, and MAFG-DT-LNC were merely reported once in BC predictive signatures, like immune-related lncRNA signature [39], epithelial mesenchymal transition-related lncRNA signature [40], tumor-infiltrating B lymphocytes lncRNA signature [41]. PSMB8-AS1 has been studied in other malignancies. Zhang and Giulietti reported that lncRNA PSMB8-AS1 could contributed to pancreatic cancer progression via modulating miR-382-3p/STAT1/PD-L1 axis and might enable to serve as a prognostic biomarker for pancreatic cancer [42,43]. Shen once reported that ELK-1 could activate PSMB8-AS1, which could modulate miR-574-5p/RAB10 and promote cell proliferation in glioma. As for AL162586.1, AP003352.1, AC016957.2, and STAG3L5P-PVRIG2P-PILRB, there have been no related studies reported to date.

We further verified the accuracy of this hypoxia lncRNA signature in subgroups stratified by clinicopathological parameters and found that, to a large extent, it could still distinguish the survival difference for patients in the high- and low risk groups (Figure 3). As for the subgroups of age ≤ 60 (*p* = 1.286e−01), metastasis subgroup (*p* = 2.802e−01) and low pathological grade (*p* = 1e+00), limited sample size in the subgroups might result in an underestimated accuracy of the hypoxia lncRNA signature to distinguish the patients with different risk scores. Moreover, we compared the risk scores in different subgroups stratified by clinical parameters. It was worth noting that the subgroups with higher pathological stages or grades usually had higher risk scores, which illustrated that our molecular signature closely related to pathological parameters and had the potential to be an important prognostic indicator (Figure 4).

Immune cells infiltration and immune checkpoint in tumor tissue played a significant role in promoting or prevent the proliferation, invasion, and migration of cancer cells, so that the immunotherapy have become a new management in cancer, such as urothelial carcinoma [8,12]. To explore the relationships between the hypoxia lncRNA signature and immune cells infiltration, we compared the immune cells content of different risk score groups and found that the contents of CD8 + T cells, activated memory CD4 + T cells, follicular helper T cells, and regulatory T cells were significantly higher in the low-risk group, while the contents of eosinophils and neutrophils were much higher in the high risk group. Recent researches reported that CD4 + T lymphocytes and CD8 + T lymphocytes infiltrating in tumor could improve the efficacy of tumor-targeted vaccine or adoptive immune cell therapy, which had been proved effective in patients with melanoma, head and neck cancer, breast cancer, and lung cancer [44–46]. Moreover, Jóźwicki once reported that BC patients with decreased infiltration of CD4 + T cells had shorter OS and CD4 + T cells were an important prognostic index of BC [47]. Follicular helper T cells signature signified organized antitumor immunity and could robustly predicted better survival or preoperative response to chemotherapy [48]. All the three immune infiltrating cells discussed above played significant roles in immune-related antitumor effect and were significantly infiltrated into tumor microenvironment of patients with low risk scores, which also demonstrated close relationship between hypoxia lncRNA and infiltrating immune cells. It was believed that regulatory T cells (Tregs) were a type of immunosuppressive cells and could potentially regulate invasiveness in BC [49]. Unexpectedly, Treg cells significantly infiltrated into tumor microenvironment of patients with low-risk scores according to the findings of our study. Whether there was a potential certain relationship between hypoxia level and Treg cells in microenvironment of BC remained unclear. Similarly, eosinophils and neutrophils infiltrating in tumor microenvironment were seldomly studied in BC.

It was demonstrated that TMB was closely related to the immunotherapy response [50,51]. The more the mutational genes existed in tumor cells, the more the mutation-associated RNA and protein might be generated, which could be recognized and targeted by immune system [52]. In our study, we listed the top 20 mutational genes in the low- and high-risk groups, and then compared risk scores between the wild types and the mutation types of top mutational genes. We found that the risk scores in the wild type of FGFR3 were significantly higher than that in the mutation type. While the risk score in the mutational type of TP53 and RB1 was higher than that in the wild type (Figure 5). FGFR3 was a carcinogenic driver and the mutation, activation, and overexpression of FGFR3 was common in BC [53,54]. Ahmad once explored the frequency of FGFR3 mutation in Indian BC patients and found that FGFR3 mutations were more common in earlier pathological stage and low-grade tumors [55]. TP53 and RB1 played a role of suppressor genes in all cancer, which encoded p53 and rb1 protein involved in regulating numerous target genes. Mutations in TP53 and RB1 were frequently observed and were closely related with poor prognosis of patients with BC [7,56,57]. Taken together, the effects caused by mutation of TP53, RB1, and FGFR3 were in accordance with the risks predicted by hypoxia risk scores in our study.

Furthermore, we enriched different genes in high- and low-risk groups and found that cancer associated pathway were enriched in the high-risk group, while the immune-related pathway belonged to the low-risk group. The cancer-associated pathways were enriched in the high-risk group, which might imply poor prognosis of patients with high score, and the immune response associated pathways in the low-risk groups usually suggested an improved prognosis. Similarly, hypoxia lncRNA-correlated mRNA functional enrichment analysis showed that metabolism and biosynthesis pathways were enriched, which were in concordance with hypoxia and reprogramming.

Along with the rapid development of drug delivery vehicles, an increasing number of ingredients were used in clinical anticancer, including chemical drugs, immune drugs, and even some fungal-derived materials [58]. Finally, we explored the signature’s predictive ability of chemotherapy and immunotherapy response and found that patients with higher risk scores tended to have lower response rates of camptothecin, vinblastine, and methotrexate. Conversely, application of cisplatin, docetaxel and thapsigargin could be more beneficial for patients with higher risk scores. Next, TMB, PD-L1/PD-L2, MSI, and IPS were employed to evaluate patients with different risk scores, and it showed that patients with higher risk scores tended to have worse immunotherapy response. Taken together, our hypoxia-associated lncRNA signature could accurately predict chemotherapy and immunotherapy response in patients with BC, which may be helpful for clinical medical decision-making. Since development and validation of the signature in our study was based on TCGA database and TCIA database, it has not been verified in large-scale clinical samples. However, we believe that with the wide application of NGS and intelligent equipment in clinic practice, noncoding RNA detection based on tumor samples will become more and more feasible [59].

## Conclusions

This is the first hypoxia lncRNA-related signature in BC, which could accurately predict the overall survival in patients with BC compared with the traditional pathological parameters. Moreover, the molecular signature has close relationships with clinicalpathological parameters, some certain infiltrating immune cells, and mutational genes in tumors. More verifications are required in future to validate the stability and practicability of the present signature.

## Supplementary Material

Supplemental Material

## Data Availability

The datasets generated and analyzed during the current study are obtained from TCGA (https://portal.gdc.cancer.gov) and the Molecular Signatures Database (https://www.gsea-msigdb.org/gsea/msigdb/index.jsp).

## Research Highlights

1. A hypoxia 10-lncRNA signature was established to predict BC patients’ prognosis.
2. The signature could predict chemotherapy and immunotherapy response accurately.
3. Functional enrichment analysis revealed potential effects of 10 lncRNAs in BC.

## References

[1] Berdik C. Unlocking bladder cancer. Nature. 2017;551(7679):S34–S5. 29117159 10.1038/551S34a

[2] Babjuk M, Burger M, Comperat EM, et al. European association of urology guidelines on non-muscle-invasive bladder cancer (TaT1 and carcinoma in situ) - 2019 update. Eur Urol. 2019;76(5):639–657. 31443960 10.1016/j.eururo.2019.08.016

[3] Witjes JA, Bruins HM, Cathomas R, et al. European association of urology guidelines on muscle-invasive and metastatic bladder cancer: summary of the 2020 guidelines. Eur Urol. 2021;79(1):82–104. 32360052 10.1016/j.eururo.2020.03.055

[4] Grayson M. Bladder cancer. Nature. 2017;551(7679):S33–S. 29117156 10.1038/551S33a

[5] Sun Z, Jing C, Xiao C, et al. An autophagy-related long non-coding RNA prognostic signature accurately predicts survival outcomes in bladder urothelial carcinoma patients. Aging (Albany NY). 2020;12(15):15624–15637. 32805727 10.18632/aging.103718 PMC7467376

[6] Robertson AG, Kim J, Al-Ahmadie H, et al. Comprehensive molecular characterization of muscle-invasive bladder cancer. Cell. 2017;171(3):540–56.e25. 28988769 10.1016/j.cell.2017.09.007 PMC5687509

[7] McConkey DJ, Choi W. Molecular subtypes of bladder cancer. Curr Oncol Rep. 2018;20(10):77. 30128829 10.1007/s11912-018-0727-5

[8] Petrova V, Annicchiarico-Petruzzelli M, Melino G, et al. The hypoxic tumour microenvironment. Oncogenesis. 2018;7(1):10. 29362402 10.1038/s41389-017-0011-9 PMC5833859

[9] Gilkes DM, Semenza GL, Wirtz D. Hypoxia and the extracellular matrix: drivers of tumour metastasis. Nat Rev Cancer. 2014;14(6):430–439. 24827502 10.1038/nrc3726 PMC4283800

[10] Rankin EB, Giaccia AJ. Hypoxic control of metastasis. Science. 2016;352(6282):175–180. 27124451 10.1126/science.aaf4405 PMC4898055

[11] Mo Z, Yu L, Cao Z, et al. Identification of a hypoxia-associated signature for lung adenocarcinoma. Front Genet. 2020;11:647. 32655624 10.3389/fgene.2020.00647 PMC7324800

[12] Liu Y, Wu J, Huang W, et al. Development and validation of a hypoxia-immune-based microenvironment gene signature for risk stratification in gastric cancer. J Transl Med. 2020;18(1):201. 32410620 10.1186/s12967-020-02366-0 PMC7226948

[13] Lin W, Wu S, Chen X, et al. Characterization of hypoxia signature to evaluate the tumor immune microenvironment and predict prognosis in glioma groups. Front Oncol. 2020;10:796. 32500034 10.3389/fonc.2020.00796 PMC7243125

[14] Zou Y-F, Rong Y-M, Tan Y-X, et al. A signature of hypoxia-related factors reveals functional dysregulation and robustly predicts clinical outcomes in stage I/II colorectal cancer patients. Cancer Cell Int. 2019;19(1):243. 31572060 10.1186/s12935-019-0964-1 PMC6757395

[15] Zhang B, Tang B, Gao J, et al. A hypoxia-related signature for clinically predicting diagnosis, prognosis and immune microenvironment of hepatocellular carcinoma patients. J Transl Med. 2020;18(1):342. 32887635 10.1186/s12967-020-02492-9 PMC7487492

[16] Boström PJ, Thoms J, Sykes J, et al. Hypoxia marker GLUT-1 (glucose transporter 1) is an independent prognostic factor for survival in bladder cancer patients treated with radical cystectomy. Bladder Cancer. 2016;2(1):101–109. 27376131 10.3233/BLC-150033 PMC4927886

[17] Hoskin PJ, Sibtain A, Daley FM, et al. GLUT1 and CAIX as intrinsic markers of hypoxia in bladder cancer: relationship with vascularity and proliferation as predictors of outcome of ARCON. Br J Cancer. 2003;89(7):1290–1297. 14520462 10.1038/sj.bjc.6601260 PMC2394309

[18] Quan J, Pan X, Zhao L, et al. LncRNA as a diagnostic and prognostic biomarker in bladder cancer: a systematic review and meta-analysis. Onco Targets Ther. 2018;11:6415–6424. 30323619 10.2147/OTT.S167853 PMC6177400

[19] Deng S-J, Chen H-Y, Ye Z, et al. Hypoxia-induced LncRNA-BX111 promotes metastasis and progression of pancreatic cancer through regulating ZEB1 transcription. Oncogene. 2018;37(44):5811–5828. 29970904 10.1038/s41388-018-0382-1

[20] Hua Q, Mi B, Xu F, et al. Hypoxia-induced lncRNA-AC020978 promotes proliferation and glycolytic metabolism of non-small cell lung cancer by regulating PKM2/HIF-1α axis. Theranostics. 2020;10(11):4762–4778. 32308748 10.7150/thno.43839 PMC7163453

[21] Liang Y, Song X, Li Y, et al. LncRNA BCRT1 promotes breast cancer progression by targeting miR-1303/PTBP3 axis. Mol Cancer. 2020;19(1):85. 32384893 10.1186/s12943-020-01206-5 PMC7206728

[22] Sun L, Wang L, Chen T, et al. LncRNA RUNX1-IT1 which is downregulated by hypoxia-driven histone deacetylase 3 represses proliferation and cancer stem-like properties in hepatocellular carcinoma cells. Cell Death Dis. 2020;11(2):95. 32024815 10.1038/s41419-020-2274-x PMC7002583

[23] Zhang J, Jin HY, Wu Y, et al. Hypoxia-induced LncRNA PCGEM1 promotes invasion and metastasis of gastric cancer through regulating SNAI1. Clin Transl Oncol. 2019;21(9):1142–1151. 30690667 10.1007/s12094-019-02035-9

[24] Cao H-L, Liu Z-J, Huang P-L, et al. lncRNA-RMRP promotes proliferation, migration and invasion of bladder cancer via miR-206. Eur Rev Med Pharmacol Sci. 2019;23(3):1012–1021. 30779067 10.26355/eurrev_201902_16988

[25] Dai G, Huang C, Yang J, et al. LncRNA SNHG3 promotes bladder cancer proliferation and metastasis through miR-515-5p/GINS2 axis. J Cell Mol Med. 2020;24(16):9231–9243. 32596993 10.1111/jcmm.15564 PMC7417716

[26] Rui X, Wang L, Pan H, et al. LncRNA GAS6-AS2 promotes bladder cancer proliferation and metastasis via GAS6-AS2/miR-298/CDK9 axis. J Cell Mol Med. 2019;23(2):865–876. 30394665 10.1111/jcmm.13986 PMC6349183

[27] Zhang C, Wang W, Lin J, et al. lncRNA CCAT1 promotes bladder cancer cell proliferation, migration and invasion. Int Braz J Urol. 2019;45(3):549–559. 31038865 10.1590/S1677-5538.IBJU.2018.0450 PMC6786104

[28] Zhuang C, Ma Q, Zhuang C, et al. LncRNA GClnc1 promotes proliferation and invasion of bladder cancer through activation of MYC. Faseb J. 2019;33(10):11045–11059. 31298933 10.1096/fj.201900078RR

[29] Chen C, He W, Huang J, et al. LNMAT1 promotes lymphatic metastasis of bladder cancer via CCL2 dependent macrophage recruitment. Nat Commun. 2018;9(1):3826. 30237493 10.1038/s41467-018-06152-x PMC6148066

[30] Chen C, Luo Y, He W, et al. Exosomal long noncoding RNA LNMAT2 promotes lymphatic metastasis in bladder cancer. J Clin Invest. 2020;130(1):404–421. 31593555 10.1172/JCI130892 PMC6934220

[31] Wang X, Li T. Development of a 15-gene signature for predicting prognosis in advanced colorectal cancer. Bioengineered. 2020;11(1):165–174. 32036725 10.1080/21655979.2020.1718459 PMC7039649

[32] Zhang F, Wang X, Bai Y, et al. Development and validation of a hypoxia-related signature for predicting survival outcomes in patients with bladder cancer. Frontiers in genetics. 2021, 12:670384. 10.3389/fgene.2021.670384 PMC8188560 34122523

[33] Wu S, Li X. A genomic instability-derived risk index predicts clinical outcome and immunotherapy response for clear cell renal cell carcinoma. Bioengineered. 2021;12(1):1642–1662. 33955826 10.1080/21655979.2021.1922330 PMC8806326

[34] Yang W, Soares J, Greninger P, et al. Genomics of drug sensitivity in cancer (GDSC): a resource for therapeutic biomarker discovery in cancer cells. Nucleic Acids Research. 2013;41(Database issue):D955–61. 23180760 10.1093/nar/gks1111 PMC3531057

[35] Shum B, Larkin J, Turajlic S. Predictive biomarkers for response to immune checkpoint inhibition. Seminars in cancer biology. 2021. 10.1016/j.semcancer.2021.03.036 33819567

[36] Charoentong P, Finotello F, Angelova M, et al. Pan-cancer immunogenomic analyses reveal genotype-immunophenotype relationships and predictors of response to checkpoint blockade. Cell Reports. 2017;18(1):248–262. 10.1016/j.celrep.2016.12.019 28052254

[37] Steinberg RL, Thomas LJ, Brooks N, et al. Multi-institution evaluation of sequential gemcitabine and docetaxel as rescue therapy for nonmuscle invasive bladder cancer. J Urol. 2020;203(5):902–909. 31821066 10.1097/JU.0000000000000688

[38] Siegel RL, Miller KD, Jemal A. Cancer Statistics, 2017. CA Cancer J Clin. 2017;67(1):7–30. 28055103 10.3322/caac.21387

[39] Wang J, Shen C, Dong D, et al. Identification and verification of an immune-related lncRNA signature for predicting the prognosis of patients with bladder cancer. International immunopharmacology. 2021, 90: 107146. 10.1016/j.intimp.2020.107146 33189610

[40] Tong H, Li T, Gao S, et al. An epithelial-mesenchymal transition-related long noncoding rna signature correlates with the prognosis and progression in bladder cancer patients. Bioscience reports. 2021, 41(1). 10.1042/BSR20203944 PMC7786330 33289830

[41] Zhou M, Zhang Z, Bao S, et al. Computational recognition of lncRNA signature of tumor-infiltrating B lymphocytes with potential implications in prognosis and immunotherapy of bladder cancer. Brief Bioinform. 2020, 22(3). 10.1093/bib/bbaa047 32382761

[42] Zhang H, Zhu C, He Z, et al. LncRNA PSMB8-AS1 contributes to pancreatic cancer progression via modulating miR-382-3p/STAT1/PD-L1 axis. J Exp Clin Cancer Res. 2020;39(1):179. 32891166 10.1186/s13046-020-01687-8 PMC7487636

[43] Giulietti M, Righetti A, Principato G, et al. LncRNA co-expression network analysis reveals novel biomarkers for pancreatic cancer. Carcinogenesis. 2018;39(8):1016–1025. 29796634 10.1093/carcin/bgy069

[44] Feldman SA, Assadipour Y, Kriley I, et al. Adoptive cell therapy--tumor-infiltrating lymphocytes, T-cell receptors, and chimeric antigen receptors. Semin Oncol. 2015;42(4):626–639. 26320066 10.1053/j.seminoncol.2015.05.005 PMC6295669

[45] Stroncek DF, Reddy O, Highfill S, et al. Advances in T-cell immunotherapies. Hematol Oncol Clin North Am. 2019;33(5):825–837. 31466607 10.1016/j.hoc.2019.05.006

[46] Arora S, Velichinskii R, Lesh RW, et al. Existing and emerging biomarkers for immune checkpoint immunotherapy in solid tumors. Adv Ther. 2019;36(10):2638–2678. 31410780 10.1007/s12325-019-01051-z PMC6778545

[47] Jóźwicki W, Brożyna AA, Siekiera J, et al. Frequency of CD4+CD25+Foxp3+ cells in peripheral blood in relation to urinary bladder cancer malignancy indicators before and after surgical removal. Oncotarget. 2016;7(10):11450–11462. 26862849 10.18632/oncotarget.7199 PMC4905485

[48] Gu-Trantien C, Loi S, Garaud S, et al. CD4⁺ follicular helper T cell infiltration predicts breast cancer survival. J Clin Invest. 2013;123(7):2873–2892. 23778140 10.1172/JCI67428 PMC3696556

[49] Winerdal ME, Krantz D, Hartana CA, et al. Urinary bladder cancer tregs suppress MMP2 and potentially regulate invasiveness. Cancer Immunol Res. 2018;6(5):528–538. 29588320 10.1158/2326-6066.CIR-17-0466

[50] June CH, O’Connor RS, Kawalekar OU, et al. CAR T cell immunotherapy for human cancer. Science. 2018;359(6382):1361–1365. 29567707 10.1126/science.aar6711

[51] Hugo W, Zaretsky JM, Sun L, et al. Genomic and transcriptomic features of response to anti-PD-1 therapy in metastatic melanoma. Cell. 2017;168(3):542. 10.1016/j.cell.2017.01.010 28129544

[52] Rizvi NA, Hellmann MD, Snyder A, et al. Cancer immunology. Mutational landscape determines sensitivity to PD-1 blockade in non-small cell lung cancer. Science. 2015;348(6230):124–128. 25765070 10.1126/science.aaa1348 PMC4993154

[53] Pouessel D, Neuzillet Y, Mertens LS, et al. Tumor heterogeneity of fibroblast growth factor receptor 3 (FGFR3) mutations in invasive bladder cancer: implications for perioperative anti-FGFR3 treatment. Ann Oncol. 2016;27(7):1311–1316. 27091807 10.1093/annonc/mdw170 PMC6608613

[54] Guancial EA, Werner L, Bellmunt J, et al. FGFR3 expression in primary and metastatic urothelial carcinoma of the bladder. Cancer Med. 2014;3(4):835–844. 24846059 10.1002/cam4.262 PMC4303151

[55] Ahmad F, Mahal V, Verma G, et al. Molecular investigation of FGFR3 gene mutation and its correlation with clinicopathological findings in Indian bladder cancer patients. Cancer Rep (Hoboken). 2018;1(3):e1130. 32721083 10.1002/cnr2.1130 PMC7941566

[56] Ciccarese C, Massari F, Blanca A, et al. Tp53 and its potential therapeutic role as a target in bladder cancer. Expert Opin Ther Targets. 2017;21(4):401–414. 28281901 10.1080/14728222.2017.1297798

[57] Wu G, Wang F, Li K, et al. Significance of TP53 mutation in bladder cancer disease progression and drug selection. PeerJ. 2019;7:e8261. 31871844 10.7717/peerj.8261 PMC6921983

[58] How CW, Ong YS, Low SS, et al. How far have we explored fungi to fight cancer? Semin Cancer Biol. 2021. DOI:10.1016/j.semcancer.2021.03.009. 33737109

[59] Shin Low S, Pan Y, Ji D, et al. Smartphone-based portable electrochemical biosensing system for detection of circulating microRNA-21 in saliva as a proof-of-concept. Sens Actuators B Chem. 2020;308:127718.

