# Supplementary material for: A hypoxia related long non-coding RNA signature could accurately predict survival outcomes in patients with bladder cancer
Source: Bioengineered. 2021 Jul 19;12(1):3802–23. doi: 10.1080/21655979.2021.1948781 (PMC8806425; doi:10.1080/21655979.2021.1948781)
Supplement: Supplemental Material [file KBIE_A_1948781_SM4898.zip › supplementary/Supplementary Table 1.docx]

Supplementary Table 1. Hypoxia genes expressed in TCGA-BLCA database

| Hypoxia_Gene |  |  |  |
| --- | --- | --- | --- |
| ACKR3 |  |  |  |
| ADM |  |  |  |
| ADORA2B |  |  |  |
| AK4 |  |  |  |
| AKAP12 |  |  |  |
| ALDOA |  |  |  |
| ALDOB |  |  |  |
| ALDOC |  |  |  |
| AMPD3 |  |  |  |
| ANGPTL4 |  |  |  |
| ANKZF1 |  |  |  |
| ANXA2 |  |  |  |
| ATF3 |  |  |  |
| ATP7A |  |  |  |
| B3GALT6 |  |  |  |
| B4GALNT2 |  |  |  |
| BCAN |  |  |  |
| BCL2 |  |  |  |
| BGN |  |  |  |
| BHLHE40 |  |  |  |
| BNIP3L |  |  |  |
| BRS3 |  |  |  |
| BTG1 |  |  |  |
| CA12 |  |  |  |
| CASP6 |  |  |  |
| CAV1 |  |  |  |
| CAVIN1 |  |  |  |
| CAVIN3 |  |  |  |
| CCNG2 |  |  |  |
| CDKN1A |  |  |  |
| CDKN1B |  |  |  |
| CDKN1C |  |  |  |
| CHST2 |  |  |  |
| CHST3 |  |  |  |
| CITED2 |  |  |  |
| COL5A1 |  |  |  |
| CP |  |  |  |
| CSRP2 |  |  |  |
| CXCR4 |  |  |  |
| DCN |  |  |  |
| DDIT3 |  |  |  |
| DDIT4 |  |  |  |
| DPYSL4 |  |  |  |
| DTNA |  |  |  |
| DUSP1 |  |  |  |
| EDN2 |  |  |  |
| EFNA1 |  |  |  |
| EFNA3 |  |  |  |
| EGFR |  |  |  |
| ENO1 |  |  |  |
| ENO2 |  |  |  |
| ENO3 |  |  |  |
| ERO1A |  |  |  |
| ERRFI1 |  |  |  |
| ETS1 |  |  |  |
| EXT1 |  |  |  |
| F3 |  |  |  |
| FAM162A |  |  |  |
| FBP1 |  |  |  |
| FOS |  |  |  |
| FOSL2 |  |  |  |
| FOXO3 |  |  |  |
| GAA |  |  |  |
| GALK1 |  |  |  |
| GAPDH |  |  |  |
| GAPDHS |  |  |  |
| GBE1 |  |  |  |
| GCK |  |  |  |
| GCNT2 |  |  |  |
| GLRX |  |  |  |
| GPC1 |  |  |  |
| GPC3 |  |  |  |
| GPC4 |  |  |  |
| GPI |  |  |  |
| GRHPR |  |  |  |
| GYS1 |  |  |  |
| HAS1 |  |  |  |
| HDLBP |  |  |  |
| HEXA |  |  |  |
| HK1 |  |  |  |
| HK2 |  |  |  |
| HMOX1 |  |  |  |
| HOXB9 |  |  |  |
| HS3ST1 |  |  |  |
| HSPA5 |  |  |  |
| IDS |  |  |  |
| IER3 |  |  |  |
| IGFBP1 |  |  |  |
| IGFBP3 |  |  |  |
| IL6 |  |  |  |
| ILVBL |  |  |  |
| INHA |  |  |  |
| IRS2 |  |  |  |
| ISG20 |  |  |  |
| JMJD6 |  |  |  |
| JUN |  |  |  |
| KDELR3 |  |  |  |
| KDM3A |  |  |  |
| KIF5A |  |  |  |
| KLF6 |  |  |  |
| KLF7 |  |  |  |
| KLHL24 |  |  |  |
| LALBA |  |  |  |
| LARGE1 |  |  |  |
| LDHA |  |  |  |
| LDHC |  |  |  |
| LOX |  |  |  |
| LXN |  |  |  |
| MAFF |  |  |  |
| MAP3K1 |  |  |  |
| MIF |  |  |  |
| MT1E |  |  |  |
| MT2A |  |  |  |
| MXI1 |  |  |  |
| MYH9 |  |  |  |
| NAGK |  |  |  |
| NCAN |  |  |  |
| NDRG1 |  |  |  |
| NDST1 |  |  |  |
| NDST2 |  |  |  |
| NEDD4L |  |  |  |
| NFIL3 |  |  |  |
| NOCT |  |  |  |
| NR3C1 |  |  |  |
| P4HA1 |  |  |  |
| P4HA2 |  |  |  |
| PAM |  |  |  |
| PCK1 |  |  |  |
| PDGFB |  |  |  |
| PDK1 |  |  |  |
| PDK3 |  |  |  |
| PFKFB3 |  |  |  |
| PFKL |  |  |  |
| PFKP |  |  |  |
| PGF |  |  |  |
| PGK1 |  |  |  |
| PGM1 |  |  |  |
| PGM2 |  |  |  |
| PHKG1 |  |  |  |
| PIM1 |  |  |  |
| PKLR |  |  |  |
| PKP1 |  |  |  |
| PLAC8 |  |  |  |
| PLAUR |  |  |  |
| PLIN2 |  |  |  |
| PNRC1 |  |  |  |
| PPARGC1A |  |  |  |
| PPFIA4 |  |  |  |
| PPP1R15A |  |  |  |
| PPP1R3C |  |  |  |
| PRDX5 |  |  |  |
| PRKCA |  |  |  |
| PYGM |  |  |  |
| RORA |  |  |  |
| RRAGD |  |  |  |
| S100A4 |  |  |  |
| SAP30 |  |  |  |
| SCARB1 |  |  |  |
| SDC2 |  |  |  |
| SDC3 |  |  |  |
| SDC4 |  |  |  |
| SELENBP1 |  |  |  |
| SERPINE1 |  |  |  |
| SIAH2 |  |  |  |
| SLC25A1 |  |  |  |
| SLC2A1 |  |  |  |
| SLC2A3 |  |  |  |
| SLC2A5 |  |  |  |
| SLC37A4 |  |  |  |
| SLC6A6 |  |  |  |
| SRPX |  |  |  |
| STBD1 |  |  |  |
| STC1 |  |  |  |
| STC2 |  |  |  |
| SULT2B1 |  |  |  |
| TES |  |  |  |
| TGFB3 |  |  |  |
| TGFBI |  |  |  |
| TGM2 |  |  |  |
| TIPARP |  |  |  |
| TKTL1 |  |  |  |
| TMEM45A |  |  |  |
| TNFAIP3 |  |  |  |
| TPBG |  |  |  |
| TPD52 |  |  |  |
| TPI1 |  |  |  |
| TPST2 |  |  |  |
| UGP2 |  |  |  |
| VEGFA |  |  |  |
| VHL |  |  |  |
| VLDLR |  |  |  |
| WSB1 |  |  |  |
| XPNPEP1 |  |  |  |
| ZFP36 |  |  |  |
| ZNF292 |  |  |  |
