# Supplementary material for: A hypoxia related long non-coding RNA signature could accurately predict survival outcomes in patients with bladder cancer
Source: Bioengineered. 2021 Jul 19;12(1):3802–23. doi: 10.1080/21655979.2021.1948781 (PMC8806425; doi:10.1080/21655979.2021.1948781)
Supplement: Supplemental Material [file KBIE_A_1948781_SM4898.zip › supplementary/Supplementary Table 2.docx]

Supplementary Table 2. Hypoxia related lncRNAs

| Hypoxia_lncRNA | Correlation_R | P_value |
| --- | --- | --- |
| AC106771.1 | >0.3 | <0.05 |
| BACE1-AS | >0.3 | <0.05 |
| AC021078.1 | >0.3 | <0.05 |
| LINC02100 | >0.3 | <0.05 |
| AC020663.2 | >0.3 | <0.05 |
| AL035563.1 | >0.3 | <0.05 |
| SEMA3B-AS1 | >0.3 | <0.05 |
| AC129926.1 | >0.3 | <0.05 |
| LINC00339 | >0.3 | <0.05 |
| DBH-AS1 | >0.3 | <0.05 |
| AC108010.1 | >0.3 | <0.05 |
| AC116914.2 | >0.3 | <0.05 |
| AL391427.1 | >0.3 | <0.05 |
| DIO3OS | >0.3 | <0.05 |
| AP000254.1 | >0.3 | <0.05 |
| AC084033.3 | >0.3 | <0.05 |
| AC009549.1 | >0.3 | <0.05 |
| AC099518.1 | >0.3 | <0.05 |
| AP003392.1 | >0.3 | <0.05 |
| GATA2-AS1 | >0.3 | <0.05 |
| SNHG19 | >0.3 | <0.05 |
| AC090152.1 | >0.3 | <0.05 |
| AP003419.3 | >0.3 | <0.05 |
| AC007383.2 | >0.3 | <0.05 |
| LINC02562 | >0.3 | <0.05 |
| AC010503.4 | >0.3 | <0.05 |
| AL133367.1 | >0.3 | <0.05 |
| AP001453.3 | >0.3 | <0.05 |
| AL021154.1 | >0.3 | <0.05 |
| AC131235.3 | >0.3 | <0.05 |
| AL118506.1 | >0.3 | <0.05 |
| AL109936.2 | >0.3 | <0.05 |
| AC027117.1 | >0.3 | <0.05 |
| IL10RB-DT | >0.3 | <0.05 |
| AC010331.1 | >0.3 | <0.05 |
| AC015922.2 | >0.3 | <0.05 |
| AC053503.4 | >0.3 | <0.05 |
| AL159169.2 | >0.3 | <0.05 |
| AC147067.1 | >0.3 | <0.05 |
| SNHG15 | >0.3 | <0.05 |
| FAM83A-AS1 | >0.3 | <0.05 |
| AL078581.1 | >0.3 | <0.05 |
| AC008105.3 | >0.3 | <0.05 |
| PWAR6 | >0.3 | <0.05 |
| CARD8-AS1 | >0.3 | <0.05 |
| CHKB-DT | >0.3 | <0.05 |
| LINC01578 | >0.3 | <0.05 |
| RNASEH1-AS1 | >0.3 | <0.05 |
| AL109614.1 | >0.3 | <0.05 |
| AL928654.1 | >0.3 | <0.05 |
| AC007388.1 | >0.3 | <0.05 |
| RAD51-AS1 | >0.3 | <0.05 |
| AC005726.3 | >0.3 | <0.05 |
| HIF1A-AS2 | >0.3 | <0.05 |
| AC024560.3 | >0.3 | <0.05 |
| AC011481.1 | >0.3 | <0.05 |
| AF131215.5 | >0.3 | <0.05 |
| AL355488.1 | >0.3 | <0.05 |
| LINC00899 | >0.3 | <0.05 |
| AL158212.2 | >0.3 | <0.05 |
| AC009065.4 | >0.3 | <0.05 |
| SNHG11 | >0.3 | <0.05 |
| HOXC-AS1 | >0.3 | <0.05 |
| AC019069.1 | >0.3 | <0.05 |
| AC012181.1 | >0.3 | <0.05 |
| PINK1-AS | >0.3 | <0.05 |
| AC009948.1 | >0.3 | <0.05 |
| MIR222HG | >0.3 | <0.05 |
| AC087276.1 | >0.3 | <0.05 |
| HCP5 | >0.3 | <0.05 |
| AC109460.2 | >0.3 | <0.05 |
| AL391069.3 | >0.3 | <0.05 |
| AC092611.2 | >0.3 | <0.05 |
| DNM3OS | >0.3 | <0.05 |
| AL139407.1 | >0.3 | <0.05 |
| ZNF529-AS1 | >0.3 | <0.05 |
| AC078880.3 | >0.3 | <0.05 |
| AC007228.1 | >0.3 | <0.05 |
| AL391244.1 | >0.3 | <0.05 |
| PGM5-AS1 | >0.3 | <0.05 |
| AL450384.2 | >0.3 | <0.05 |
| LINC01857 | >0.3 | <0.05 |
| HCG27 | >0.3 | <0.05 |
| SNHG25 | >0.3 | <0.05 |
| AC007406.5 | >0.3 | <0.05 |
| AC004982.2 | >0.3 | <0.05 |
| AC254562.3 | >0.3 | <0.05 |
| MIR210HG | >0.3 | <0.05 |
| AC121764.1 | >0.3 | <0.05 |
| AC004585.1 | >0.3 | <0.05 |
| LINC01004 | >0.3 | <0.05 |
| AC093388.1 | >0.3 | <0.05 |
| PAXIP1-AS2 | >0.3 | <0.05 |
| AC135178.3 | >0.3 | <0.05 |
| AGAP2-AS1 | >0.3 | <0.05 |
| AC091057.1 | >0.3 | <0.05 |
| AC012181.2 | >0.3 | <0.05 |
| SNHG6 | >0.3 | <0.05 |
| AL359091.5 | >0.3 | <0.05 |
| AC084125.4 | >0.3 | <0.05 |
| AC106782.2 | >0.3 | <0.05 |
| AC006042.1 | >0.3 | <0.05 |
| AC135178.5 | >0.3 | <0.05 |
| AC008074.2 | >0.3 | <0.05 |
| LINC01963 | >0.3 | <0.05 |
| AC105219.3 | >0.3 | <0.05 |
| PDCD4-AS1 | >0.3 | <0.05 |
| AC092171.4 | >0.3 | <0.05 |
| LINC02541 | >0.3 | <0.05 |
| LINC01564 | >0.3 | <0.05 |
| NUTM2B-AS1 | >0.3 | <0.05 |
| AC108449.2 | >0.3 | <0.05 |
| AC142472.1 | >0.3 | <0.05 |
| TSPEAR-AS1 | >0.3 | <0.05 |
| AC073842.2 | >0.3 | <0.05 |
| AL031665.2 | >0.3 | <0.05 |
| AL033397.2 | >0.3 | <0.05 |
| LINC00973 | >0.3 | <0.05 |
| AC023421.2 | >0.3 | <0.05 |
| LINC02577 | >0.3 | <0.05 |
| AL133355.1 | >0.3 | <0.05 |
| AL359962.2 | >0.3 | <0.05 |
| AC018638.7 | >0.3 | <0.05 |
| LINC-PINT | >0.3 | <0.05 |
| AC092803.2 | >0.3 | <0.05 |
| AC020594.1 | >0.3 | <0.05 |
| AC092794.1 | >0.3 | <0.05 |
| FAM13A-AS1 | >0.3 | <0.05 |
| AC068580.1 | >0.3 | <0.05 |
| AL049597.2 | >0.3 | <0.05 |
| LINC01671 | >0.3 | <0.05 |
| HOXB-AS4 | >0.3 | <0.05 |
| AC012467.2 | >0.3 | <0.05 |
| AP000442.2 | >0.3 | <0.05 |
| AC073896.3 | >0.3 | <0.05 |
| AC006001.2 | >0.3 | <0.05 |
| AC093673.1 | >0.3 | <0.05 |
| FAM160A1-DT | >0.3 | <0.05 |
| LINC02163 | >0.3 | <0.05 |
| AC090181.2 | >0.3 | <0.05 |
| AC098484.1 | >0.3 | <0.05 |
| AC026471.4 | >0.3 | <0.05 |
| ZNF426-DT | >0.3 | <0.05 |
| TMEM161B-AS1 | >0.3 | <0.05 |
| AC125807.2 | >0.3 | <0.05 |
| TBILA | >0.3 | <0.05 |
| AL354892.2 | >0.3 | <0.05 |
| CASC9 | >0.3 | <0.05 |
| AC004148.2 | >0.3 | <0.05 |
| AL596094.1 | >0.3 | <0.05 |
| AC090579.1 | >0.3 | <0.05 |
| AC005911.1 | >0.3 | <0.05 |
| MIR22HG | >0.3 | <0.05 |
| AC012615.6 | >0.3 | <0.05 |
| AC108112.1 | >0.3 | <0.05 |
| AC080129.2 | >0.3 | <0.05 |
| AL365330.1 | >0.3 | <0.05 |
| AC020913.3 | >0.3 | <0.05 |
| AP000692.1 | >0.3 | <0.05 |
| ATP2A1-AS1 | >0.3 | <0.05 |
| AC023510.2 | >0.3 | <0.05 |
| AC132192.2 | >0.3 | <0.05 |
| AC008124.1 | >0.3 | <0.05 |
| AC074032.1 | >0.3 | <0.05 |
| AC018647.2 | >0.3 | <0.05 |
| EPB41L4A-AS1 | >0.3 | <0.05 |
| AC011503.2 | >0.3 | <0.05 |
| PRR34-AS1 | >0.3 | <0.05 |
| AL136084.3 | >0.3 | <0.05 |
| SH3PXD2A-AS1 | >0.3 | <0.05 |
| ZNF433-AS1 | >0.3 | <0.05 |
| LINC01431 | >0.3 | <0.05 |
| DGCR11 | >0.3 | <0.05 |
| AC027031.2 | >0.3 | <0.05 |
| AP005432.2 | >0.3 | <0.05 |
| AC005840.4 | >0.3 | <0.05 |
| AC063948.1 | >0.3 | <0.05 |
| AP003392.4 | >0.3 | <0.05 |
| AC107068.1 | >0.3 | <0.05 |
| AC068580.3 | >0.3 | <0.05 |
| AC011472.1 | >0.3 | <0.05 |
| AC124045.1 | >0.3 | <0.05 |
| AC097103.2 | >0.3 | <0.05 |
| AC007342.4 | >0.3 | <0.05 |
| AL021068.1 | >0.3 | <0.05 |
| LINC01614 | >0.3 | <0.05 |
| BHLHE40-AS1 | >0.3 | <0.05 |
| AC005785.1 | >0.3 | <0.05 |
| LINC01287 | >0.3 | <0.05 |
| AC116407.2 | >0.3 | <0.05 |
| AC015912.3 | >0.3 | <0.05 |
| AC002470.1 | >0.3 | <0.05 |
| ZNF460-AS1 | >0.3 | <0.05 |
| DNAJC3-DT | >0.3 | <0.05 |
| RPARP-AS1 | >0.3 | <0.05 |
| AC127024.5 | >0.3 | <0.05 |
| AC008764.6 | >0.3 | <0.05 |
| AC008969.1 | >0.3 | <0.05 |
| TMPO-AS1 | >0.3 | <0.05 |
| AC066613.1 | >0.3 | <0.05 |
| OSER1-DT | >0.3 | <0.05 |
| AC234582.1 | >0.3 | <0.05 |
| AL139287.1 | >0.3 | <0.05 |
| AC012236.1 | >0.3 | <0.05 |
| AC009779.2 | >0.3 | <0.05 |
| LINC00665 | >0.3 | <0.05 |
| AP005230.1 | >0.3 | <0.05 |
| MID1IP1-AS1 | >0.3 | <0.05 |
| AC010186.3 | >0.3 | <0.05 |
| AC005034.5 | >0.3 | <0.05 |
| IPO5P1 | >0.3 | <0.05 |
| AC012368.1 | >0.3 | <0.05 |
| RBPMS-AS1 | >0.3 | <0.05 |
| AC134312.5 | >0.3 | <0.05 |
| AL138756.1 | >0.3 | <0.05 |
| PPP1R26-AS1 | >0.3 | <0.05 |
| AL356740.1 | >0.3 | <0.05 |
| AC020978.4 | >0.3 | <0.05 |
| NCK1-DT | >0.3 | <0.05 |
| AP006621.3 | >0.3 | <0.05 |
| AP002748.3 | >0.3 | <0.05 |
| SBF2-AS1 | >0.3 | <0.05 |
| AL049840.3 | >0.3 | <0.05 |
| AL513329.1 | >0.3 | <0.05 |
| DUXAP8 | >0.3 | <0.05 |
| LINC00460 | >0.3 | <0.05 |
| AC104785.1 | >0.3 | <0.05 |
| AL513320.1 | >0.3 | <0.05 |
| AL024508.1 | >0.3 | <0.05 |
| RASSF8-AS1 | >0.3 | <0.05 |
| AC024361.1 | >0.3 | <0.05 |
| AC009120.3 | >0.3 | <0.05 |
| AC008115.3 | >0.3 | <0.05 |
| AC022306.2 | >0.3 | <0.05 |
| AC080013.1 | >0.3 | <0.05 |
| LINC02518 | >0.3 | <0.05 |
| CIRBP-AS1 | >0.3 | <0.05 |
| AC091271.1 | >0.3 | <0.05 |
| AC105020.1 | >0.3 | <0.05 |
| AC022007.1 | >0.3 | <0.05 |
| AC011462.4 | >0.3 | <0.05 |
| AC039056.2 | >0.3 | <0.05 |
| AC124312.5 | >0.3 | <0.05 |
| AL021707.6 | >0.3 | <0.05 |
| AC080013.6 | >0.3 | <0.05 |
| AL121603.2 | >0.3 | <0.05 |
| LINC01605 | >0.3 | <0.05 |
| AC097376.2 | >0.3 | <0.05 |
| AC006547.1 | >0.3 | <0.05 |
| BRWD1-AS2 | >0.3 | <0.05 |
| CD44-AS1 | >0.3 | <0.05 |
| PCED1B-AS1 | >0.3 | <0.05 |
| AC073046.1 | >0.3 | <0.05 |
| AL353804.1 | >0.3 | <0.05 |
| LINC01094 | >0.3 | <0.05 |
| MIF-AS1 | >0.3 | <0.05 |
| AC022568.1 | >0.3 | <0.05 |
| MIR100HG | >0.3 | <0.05 |
| ZNF213-AS1 | >0.3 | <0.05 |
| AL451050.2 | >0.3 | <0.05 |
| AC009414.2 | >0.3 | <0.05 |
| AF131215.6 | >0.3 | <0.05 |
| AC008906.1 | >0.3 | <0.05 |
| PSPC1-AS2 | >0.3 | <0.05 |
| LINC00519 | >0.3 | <0.05 |
| AL731533.2 | >0.3 | <0.05 |
| MAPKAPK5-AS1 | >0.3 | <0.05 |
| AP002336.2 | >0.3 | <0.05 |
| B3GALT5-AS1 | >0.3 | <0.05 |
| AL139349.1 | >0.3 | <0.05 |
| LINC01176 | >0.3 | <0.05 |
| AC093110.1 | >0.3 | <0.05 |
| TNFRSF10A-AS1 | >0.3 | <0.05 |
| AC020915.2 | >0.3 | <0.05 |
| AC131532.1 | >0.3 | <0.05 |
| AL021878.2 | >0.3 | <0.05 |
| FGD5-AS1 | >0.3 | <0.05 |
| AC078909.2 | >0.3 | <0.05 |
| LINC02195 | >0.3 | <0.05 |
| AC008763.1 | >0.3 | <0.05 |
| ZNF793-AS1 | >0.3 | <0.05 |
| AC079949.2 | >0.3 | <0.05 |
| AC138207.5 | >0.3 | <0.05 |
| MIAT | >0.3 | <0.05 |
| ZFAS1 | >0.3 | <0.05 |
| MYOSLID | >0.3 | <0.05 |
| AC006329.1 | >0.3 | <0.05 |
| AL133371.2 | >0.3 | <0.05 |
| AC064836.3 | >0.3 | <0.05 |
| AL122023.1 | >0.3 | <0.05 |
| TMEM147-AS1 | >0.3 | <0.05 |
| AC005180.2 | >0.3 | <0.05 |
| AC009065.8 | >0.3 | <0.05 |
| AC245041.1 | >0.3 | <0.05 |
| ZKSCAN2-DT | >0.3 | <0.05 |
| AL358472.2 | >0.3 | <0.05 |
| FLJ37453 | >0.3 | <0.05 |
| AC010834.3 | >0.3 | <0.05 |
| AL031714.1 | >0.3 | <0.05 |
| AL513318.2 | >0.3 | <0.05 |
| AC109322.1 | >0.3 | <0.05 |
| LINC01876 | >0.3 | <0.05 |
| AL121820.2 | >0.3 | <0.05 |
| AL021807.1 | >0.3 | <0.05 |
| AC005674.1 | >0.3 | <0.05 |
| TRAM2-AS1 | >0.3 | <0.05 |
| AC107081.2 | >0.3 | <0.05 |
| AL513165.1 | >0.3 | <0.05 |
| MIR3936HG | >0.3 | <0.05 |
| APTR | >0.3 | <0.05 |
| AC026471.1 | >0.3 | <0.05 |
| AC107375.1 | >0.3 | <0.05 |
| AC110619.1 | >0.3 | <0.05 |
| AC087741.2 | >0.3 | <0.05 |
| AL604028.1 | >0.3 | <0.05 |
| AL157932.1 | >0.3 | <0.05 |
| AC091057.4 | >0.3 | <0.05 |
| AC008443.4 | >0.3 | <0.05 |
| AC079684.1 | >0.3 | <0.05 |
| AC022211.3 | >0.3 | <0.05 |
| AC006206.2 | >0.3 | <0.05 |
| AL353622.1 | >0.3 | <0.05 |
| AC010654.1 | >0.3 | <0.05 |
| ASH1L-AS1 | >0.3 | <0.05 |
| LINC00346 | >0.3 | <0.05 |
| ZNF582-AS1 | >0.3 | <0.05 |
| AL109811.2 | >0.3 | <0.05 |
| SNHG10 | >0.3 | <0.05 |
| LINC00852 | >0.3 | <0.05 |
| AC015726.1 | >0.3 | <0.05 |
| AC092484.1 | >0.3 | <0.05 |
| AC037459.3 | >0.3 | <0.05 |
| AC006942.1 | >0.3 | <0.05 |
| AC125257.1 | >0.3 | <0.05 |
| LINC00511 | >0.3 | <0.05 |
| AL391422.4 | >0.3 | <0.05 |
| AC027097.1 | >0.3 | <0.05 |
| MIR600HG | >0.3 | <0.05 |
| AC046143.1 | >0.3 | <0.05 |
| AC018904.1 | >0.3 | <0.05 |
| AP004609.3 | >0.3 | <0.05 |
| AC062017.1 | >0.3 | <0.05 |
| AC084117.1 | >0.3 | <0.05 |
| AL161668.4 | >0.3 | <0.05 |
| AC104041.1 | >0.3 | <0.05 |
| AL390728.6 | >0.3 | <0.05 |
| SNHG9 | >0.3 | <0.05 |
| TONSL-AS1 | >0.3 | <0.05 |
| AC009309.1 | >0.3 | <0.05 |
| AC026369.2 | >0.3 | <0.05 |
| AP006284.1 | >0.3 | <0.05 |
| EBLN3P | >0.3 | <0.05 |
| DUBR | >0.3 | <0.05 |
| AC007485.1 | >0.3 | <0.05 |
| AP000695.1 | >0.3 | <0.05 |
| AL158212.3 | >0.3 | <0.05 |
| AL096828.3 | >0.3 | <0.05 |
| AC000123.1 | >0.3 | <0.05 |
| AC090589.3 | >0.3 | <0.05 |
| AC022144.1 | >0.3 | <0.05 |
| AC008438.1 | >0.3 | <0.05 |
| AGBL5-IT1 | >0.3 | <0.05 |
| LINC00885 | >0.3 | <0.05 |
| LINC01144 | >0.3 | <0.05 |
| AP002907.1 | >0.3 | <0.05 |
| MIR548XHG | >0.3 | <0.05 |
| AC010789.1 | >0.3 | <0.05 |
| AL590723.1 | >0.3 | <0.05 |
| PSMA3-AS1 | >0.3 | <0.05 |
| AC027796.4 | >0.3 | <0.05 |
| AP006621.2 | >0.3 | <0.05 |
| AC007114.1 | >0.3 | <0.05 |
| AL353708.3 | >0.3 | <0.05 |
| MCF2L-AS1 | >0.3 | <0.05 |
| AC022211.1 | >0.3 | <0.05 |
| NNT-AS1 | >0.3 | <0.05 |
| AC015922.3 | >0.3 | <0.05 |
| AC132807.2 | >0.3 | <0.05 |
| AL121829.2 | >0.3 | <0.05 |
| AL118505.1 | >0.3 | <0.05 |
| AC005083.1 | >0.3 | <0.05 |
| LINC01518 | >0.3 | <0.05 |
| AC010275.1 | >0.3 | <0.05 |
| AC107294.2 | >0.3 | <0.05 |
| AL592211.1 | >0.3 | <0.05 |
| AC073957.3 | >0.3 | <0.05 |
| LINC01082 | >0.3 | <0.05 |
| MIR762HG | >0.3 | <0.05 |
| AC105942.1 | >0.3 | <0.05 |
| AC005009.1 | >0.3 | <0.05 |
| AL031775.1 | >0.3 | <0.05 |
| AL355987.4 | >0.3 | <0.05 |
| LINC01560 | >0.3 | <0.05 |
| AC100810.1 | >0.3 | <0.05 |
| LINC00239 | >0.3 | <0.05 |
| MIR155HG | >0.3 | <0.05 |
| DLEU1 | >0.3 | <0.05 |
| LINC01137 | >0.3 | <0.05 |
| AC116667.1 | >0.3 | <0.05 |
| ZNF503-AS2 | >0.3 | <0.05 |
| AC004943.2 | >0.3 | <0.05 |
| MSC-AS1 | >0.3 | <0.05 |
| AL513218.1 | >0.3 | <0.05 |
| AC108062.1 | >0.3 | <0.05 |
| AC005696.1 | >0.3 | <0.05 |
| AL161772.1 | >0.3 | <0.05 |
| AC130371.2 | >0.3 | <0.05 |
| DGUOK-AS1 | >0.3 | <0.05 |
| COLCA1 | >0.3 | <0.05 |
| MAGI2-AS3 | >0.3 | <0.05 |
| AC093752.3 | >0.3 | <0.05 |
| CEBPA-DT | >0.3 | <0.05 |
| AC026740.1 | >0.3 | <0.05 |
| YTHDF3-AS1 | >0.3 | <0.05 |
| AL590094.1 | >0.3 | <0.05 |
| AL691432.2 | >0.3 | <0.05 |
| AL603839.2 | >0.3 | <0.05 |
| AC080013.3 | >0.3 | <0.05 |
| AC084036.1 | >0.3 | <0.05 |
| AC139768.1 | >0.3 | <0.05 |
| AC068620.2 | >0.3 | <0.05 |
| AP000759.1 | >0.3 | <0.05 |
| AC016876.1 | >0.3 | <0.05 |
| LINC00165 | >0.3 | <0.05 |
| AC108673.3 | >0.3 | <0.05 |
| AC026801.2 | >0.3 | <0.05 |
| BX322234.1 | >0.3 | <0.05 |
| TAPT1-AS1 | >0.3 | <0.05 |
| LINC01569 | >0.3 | <0.05 |
| AC067930.5 | >0.3 | <0.05 |
| CR936218.1 | >0.3 | <0.05 |
| AC084824.5 | >0.3 | <0.05 |
| NORAD | >0.3 | <0.05 |
| LINC01816 | >0.3 | <0.05 |
| AL031667.3 | >0.3 | <0.05 |
| AC008764.2 | >0.3 | <0.05 |
| LINC01767 | >0.3 | <0.05 |
| LNCTAM34A | >0.3 | <0.05 |
| AL031775.2 | >0.3 | <0.05 |
| SNHG1 | >0.3 | <0.05 |
| LINC01355 | >0.3 | <0.05 |
| AC120498.4 | >0.3 | <0.05 |
| AC046143.2 | >0.3 | <0.05 |
| AC079610.2 | >0.3 | <0.05 |
| AL513477.2 | >0.3 | <0.05 |
| AC083862.2 | >0.3 | <0.05 |
| AC008760.2 | >0.3 | <0.05 |
| FOXCUT | >0.3 | <0.05 |
| AC115618.1 | >0.3 | <0.05 |
| AL133520.1 | >0.3 | <0.05 |
| AC114956.1 | >0.3 | <0.05 |
| AL583785.1 | >0.3 | <0.05 |
| AL139289.1 | >0.3 | <0.05 |
| AC020916.1 | >0.3 | <0.05 |
| LINC01341 | >0.3 | <0.05 |
| Z83843.1 | >0.3 | <0.05 |
| AC104667.2 | >0.3 | <0.05 |
| AP002360.3 | >0.3 | <0.05 |
| AC243960.1 | >0.3 | <0.05 |
| Z98884.2 | >0.3 | <0.05 |
| AC040169.1 | >0.3 | <0.05 |
| AP001505.1 | >0.3 | <0.05 |
| TMEM9B-AS1 | >0.3 | <0.05 |
| GHRLOS | >0.3 | <0.05 |
| AC092279.1 | >0.3 | <0.05 |
| AC104825.1 | >0.3 | <0.05 |
| POLH-AS1 | >0.3 | <0.05 |
| AL121944.1 | >0.3 | <0.05 |
| AP001625.2 | >0.3 | <0.05 |
| LINC01535 | >0.3 | <0.05 |
| AC132938.3 | >0.3 | <0.05 |
| AC022509.3 | >0.3 | <0.05 |
| AC020910.4 | >0.3 | <0.05 |
| AC145207.5 | >0.3 | <0.05 |
| AL391121.1 | >0.3 | <0.05 |
| AC019131.2 | >0.3 | <0.05 |
| LINC01376 | >0.3 | <0.05 |
| GABPB1-AS1 | >0.3 | <0.05 |
| AC027228.2 | >0.3 | <0.05 |
| ZNF32-AS2 | >0.3 | <0.05 |
| LINC002481 | >0.3 | <0.05 |
| PCAT6 | >0.3 | <0.05 |
| AC087481.3 | >0.3 | <0.05 |
| AC073896.4 | >0.3 | <0.05 |
| AC004656.1 | >0.3 | <0.05 |
| AC008870.2 | >0.3 | <0.05 |
| AC004687.1 | >0.3 | <0.05 |
| AC083843.2 | >0.3 | <0.05 |
| LINC01607 | >0.3 | <0.05 |
| AC137630.3 | >0.3 | <0.05 |
| AC012640.2 | >0.3 | <0.05 |
| AC106782.6 | >0.3 | <0.05 |
| AC025175.1 | >0.3 | <0.05 |
| AC084018.1 | >0.3 | <0.05 |
| AC022400.1 | >0.3 | <0.05 |
| AL049840.2 | >0.3 | <0.05 |
| AL390719.2 | >0.3 | <0.05 |
| AC026333.4 | >0.3 | <0.05 |
| AC127024.4 | >0.3 | <0.05 |
| AC129510.1 | >0.3 | <0.05 |
| LINC01133 | >0.3 | <0.05 |
| AL139089.1 | >0.3 | <0.05 |
| INE1 | >0.3 | <0.05 |
| AC114956.2 | >0.3 | <0.05 |
| AC025171.4 | >0.3 | <0.05 |
| AC090198.1 | >0.3 | <0.05 |
| AP000894.4 | >0.3 | <0.05 |
| TNFRSF14-AS1 | >0.3 | <0.05 |
| LINC02544 | >0.3 | <0.05 |
| SNHG26 | >0.3 | <0.05 |
| AL358472.3 | >0.3 | <0.05 |
| AC006026.3 | >0.3 | <0.05 |
| GEMIN7-AS1 | >0.3 | <0.05 |
| AL163051.1 | >0.3 | <0.05 |
| AC012306.2 | >0.3 | <0.05 |
| EMX2OS | >0.3 | <0.05 |
| MIR31HG | >0.3 | <0.05 |
| AP003071.4 | >0.3 | <0.05 |
| AL138976.2 | >0.3 | <0.05 |
| SNHG12 | >0.3 | <0.05 |
| AP006621.4 | >0.3 | <0.05 |
| IER3-AS1 | >0.3 | <0.05 |
| AC005899.6 | >0.3 | <0.05 |
| AC011461.1 | >0.3 | <0.05 |
| AP001065.1 | >0.3 | <0.05 |
| LINC00662 | >0.3 | <0.05 |
| AL355472.1 | >0.3 | <0.05 |
| AC012511.1 | >0.3 | <0.05 |
| LINC01356 | >0.3 | <0.05 |
| AC126178.1 | >0.3 | <0.05 |
| LINC01615 | >0.3 | <0.05 |
| AC005261.3 | >0.3 | <0.05 |
| AL035413.1 | >0.3 | <0.05 |
| U62317.1 | >0.3 | <0.05 |
| AC006333.2 | >0.3 | <0.05 |
| ERVE-1 | >0.3 | <0.05 |
| AC073575.2 | >0.3 | <0.05 |
| THAP9-AS1 | >0.3 | <0.05 |
| AC069307.1 | >0.3 | <0.05 |
| AC002059.1 | >0.3 | <0.05 |
| AC010273.2 | >0.3 | <0.05 |
| AC108673.2 | >0.3 | <0.05 |
| AC141002.1 | >0.3 | <0.05 |
| AC027307.2 | >0.3 | <0.05 |
| RAB30-AS1 | >0.3 | <0.05 |
| AC008608.2 | >0.3 | <0.05 |
| AL355075.4 | >0.3 | <0.05 |
| AC011468.1 | >0.3 | <0.05 |
| AL035446.1 | >0.3 | <0.05 |
| LINC00685 | >0.3 | <0.05 |
| AL022328.3 | >0.3 | <0.05 |
| HOXB-AS3 | >0.3 | <0.05 |
| AC104695.2 | >0.3 | <0.05 |
| AC090425.1 | >0.3 | <0.05 |
| JPX | >0.3 | <0.05 |
| AL513534.1 | >0.3 | <0.05 |
| AL031600.1 | >0.3 | <0.05 |
| AC090948.1 | >0.3 | <0.05 |
| AC093726.2 | >0.3 | <0.05 |
| AC027644.3 | >0.3 | <0.05 |
| AC018695.6 | >0.3 | <0.05 |
| ELF3-AS1 | >0.3 | <0.05 |
| ADNP-AS1 | >0.3 | <0.05 |
| AC012170.2 | >0.3 | <0.05 |
| LINC02038 | >0.3 | <0.05 |
| AC244153.1 | >0.3 | <0.05 |
| AC010542.5 | >0.3 | <0.05 |
| Z82243.1 | >0.3 | <0.05 |
| LINC01336 | >0.3 | <0.05 |
| AP002026.1 | >0.3 | <0.05 |
| AC020765.2 | >0.3 | <0.05 |
| AC090246.1 | >0.3 | <0.05 |
| AC009061.2 | >0.3 | <0.05 |
| CCDC183-AS1 | >0.3 | <0.05 |
| AL122125.1 | >0.3 | <0.05 |
| AC011503.1 | >0.3 | <0.05 |
| AC006270.1 | >0.3 | <0.05 |
| AC004706.1 | >0.3 | <0.05 |
| PAN3-AS1 | >0.3 | <0.05 |
| ZNF674-AS1 | >0.3 | <0.05 |
| LINC00909 | >0.3 | <0.05 |
| MIR193BHG | >0.3 | <0.05 |
| RTCA-AS1 | >0.3 | <0.05 |
| ASMTL-AS1 | >0.3 | <0.05 |
| AL360181.2 | >0.3 | <0.05 |
| LINC00930 | >0.3 | <0.05 |
| AC008035.1 | >0.3 | <0.05 |
| LINC02446 | >0.3 | <0.05 |
| ILF3-DT | >0.3 | <0.05 |
| AL021707.3 | >0.3 | <0.05 |
| AL844908.1 | >0.3 | <0.05 |
| MELTF-AS1 | >0.3 | <0.05 |
| LINC00106 | >0.3 | <0.05 |
| MAST4-AS1 | >0.3 | <0.05 |
| AP000240.1 | >0.3 | <0.05 |
| PAXIP1-AS1 | >0.3 | <0.05 |
| AC068594.1 | >0.3 | <0.05 |
| AC055822.1 | >0.3 | <0.05 |
| SNHG3 | >0.3 | <0.05 |
| AC074212.1 | >0.3 | <0.05 |
| U62317.4 | >0.3 | <0.05 |
| AL135999.3 | >0.3 | <0.05 |
| AC025287.3 | >0.3 | <0.05 |
| MEG3 | >0.3 | <0.05 |
| ZNF561-AS1 | >0.3 | <0.05 |
| AL359915.2 | >0.3 | <0.05 |
| AC084125.2 | >0.3 | <0.05 |
| AC119403.1 | >0.3 | <0.05 |
| AL121772.3 | >0.3 | <0.05 |
| AC106795.2 | >0.3 | <0.05 |
| AL117332.1 | >0.3 | <0.05 |
| AL359962.1 | >0.3 | <0.05 |
| AC004975.2 | >0.3 | <0.05 |
| USP30-AS1 | >0.3 | <0.05 |
| OTUD6B-AS1 | >0.3 | <0.05 |
| AC009690.2 | >0.3 | <0.05 |
| AC026979.2 | >0.3 | <0.05 |
| AC009812.1 | >0.3 | <0.05 |
| LINC00992 | >0.3 | <0.05 |
| LINC01547 | >0.3 | <0.05 |
| AC013652.1 | >0.3 | <0.05 |
| LINC02560 | >0.3 | <0.05 |
| AC147651.3 | >0.3 | <0.05 |
| AC034231.1 | >0.3 | <0.05 |
| RMRP | >0.3 | <0.05 |
| BX537318.1 | >0.3 | <0.05 |
| LINC01503 | >0.3 | <0.05 |
| AC007255.1 | >0.3 | <0.05 |
| FMR1-IT1 | >0.3 | <0.05 |
| AC004080.2 | >0.3 | <0.05 |
| AC002128.1 | >0.3 | <0.05 |
| AC004477.3 | >0.3 | <0.05 |
| AC004918.1 | >0.3 | <0.05 |
| ITGB2-AS1 | >0.3 | <0.05 |
| AP001318.2 | >0.3 | <0.05 |
| AC138207.4 | >0.3 | <0.05 |
| AC002128.2 | >0.3 | <0.05 |
| FLJ42351 | >0.3 | <0.05 |
| AC048341.2 | >0.3 | <0.05 |
| AC010973.2 | >0.3 | <0.05 |
| AC068338.2 | >0.3 | <0.05 |
| AL109917.1 | >0.3 | <0.05 |
| AC026304.1 | >0.3 | <0.05 |
| AC074117.1 | >0.3 | <0.05 |
| SENCR | >0.3 | <0.05 |
| AC002550.2 | >0.3 | <0.05 |
| AL354836.1 | >0.3 | <0.05 |
| CFAP58-DT | >0.3 | <0.05 |
| AF127577.4 | >0.3 | <0.05 |
| AL451085.2 | >0.3 | <0.05 |
| AC009318.4 | >0.3 | <0.05 |
| AL031123.1 | >0.3 | <0.05 |
| ENTPD3-AS1 | >0.3 | <0.05 |
| AL139286.2 | >0.3 | <0.05 |
| AC012073.1 | >0.3 | <0.05 |
| EP300-AS1 | >0.3 | <0.05 |
| AL117336.2 | >0.3 | <0.05 |
| AP001434.1 | >0.3 | <0.05 |
| AC093249.6 | >0.3 | <0.05 |
| AC011632.1 | >0.3 | <0.05 |
| LINC02593 | >0.3 | <0.05 |
| AC108865.1 | >0.3 | <0.05 |
| AC106028.3 | >0.3 | <0.05 |
| AC009237.15 | >0.3 | <0.05 |
| KCTD21-AS1 | >0.3 | <0.05 |
| AL023803.1 | >0.3 | <0.05 |
| C17orf82 | >0.3 | <0.05 |
| AC139887.2 | >0.3 | <0.05 |
| HAGLR | >0.3 | <0.05 |
| AC027020.2 | >0.3 | <0.05 |
| AL122010.1 | >0.3 | <0.05 |
| AL109613.1 | >0.3 | <0.05 |
| MAP3K14-AS1 | >0.3 | <0.05 |
| AL162258.2 | >0.3 | <0.05 |
| AC026167.1 | >0.3 | <0.05 |
| AC010636.1 | >0.3 | <0.05 |
| AL731567.1 | >0.3 | <0.05 |
| AC010615.2 | >0.3 | <0.05 |
| AC124319.1 | >0.3 | <0.05 |
| KCNMB2-AS1 | >0.3 | <0.05 |
| Z69706.1 | >0.3 | <0.05 |
| AFAP1-AS1 | >0.3 | <0.05 |
| AC015871.3 | >0.3 | <0.05 |
| AL033527.2 | >0.3 | <0.05 |
| AC018690.1 | >0.3 | <0.05 |
| RUSC1-AS1 | >0.3 | <0.05 |
| AC008687.3 | >0.3 | <0.05 |
| AL591848.4 | >0.3 | <0.05 |
| ALG13-AS1 | >0.3 | <0.05 |
| AC005104.1 | >0.3 | <0.05 |
| WNT5A-AS1 | >0.3 | <0.05 |
| AL162274.2 | >0.3 | <0.05 |
| IDH1-AS1 | >0.3 | <0.05 |
| AC004596.1 | >0.3 | <0.05 |
| AL589843.1 | >0.3 | <0.05 |
| AL008582.1 | >0.3 | <0.05 |
| AP002884.1 | >0.3 | <0.05 |
| C5orf56 | >0.3 | <0.05 |
| SH3BP5-AS1 | >0.3 | <0.05 |
| AC110285.6 | >0.3 | <0.05 |
| AC021146.12 | >0.3 | <0.05 |
| DCST1-AS1 | >0.3 | <0.05 |
| AL445524.1 | >0.3 | <0.05 |
| AL513550.1 | >0.3 | <0.05 |
| AC011472.4 | >0.3 | <0.05 |
| FLJ20021 | >0.3 | <0.05 |
| AC098487.1 | >0.3 | <0.05 |
| PIK3CD-AS2 | >0.3 | <0.05 |
| AC009812.4 | >0.3 | <0.05 |
| AL606489.1 | >0.3 | <0.05 |
| WAC-AS1 | >0.3 | <0.05 |
| AC018645.2 | >0.3 | <0.05 |
| AL031058.1 | >0.3 | <0.05 |
| AC024060.1 | >0.3 | <0.05 |
| AC061992.1 | >0.3 | <0.05 |
| ZFPM2-AS1 | >0.3 | <0.05 |
| AC105219.1 | >0.3 | <0.05 |
| FIRRE | >0.3 | <0.05 |
| AATBC | >0.3 | <0.05 |
| AC006213.4 | >0.3 | <0.05 |
| AC018653.3 | >0.3 | <0.05 |
| LINC00893 | >0.3 | <0.05 |
| AC060780.1 | >0.3 | <0.05 |
| AP005482.3 | >0.3 | <0.05 |
| AC092119.2 | >0.3 | <0.05 |
| AL596244.1 | >0.3 | <0.05 |
| TNRC6C-AS1 | >0.3 | <0.05 |
| AL133243.2 | >0.3 | <0.05 |
| AC007998.4 | >0.3 | <0.05 |
| KDM4A-AS1 | >0.3 | <0.05 |
| AL035425.3 | >0.3 | <0.05 |
| AC012146.1 | >0.3 | <0.05 |
| AC010168.2 | >0.3 | <0.05 |
| AC006262.1 | >0.3 | <0.05 |
| OVOL1-AS1 | >0.3 | <0.05 |
| AC009120.2 | >0.3 | <0.05 |
| AC104534.1 | >0.3 | <0.05 |
| AC139795.3 | >0.3 | <0.05 |
| IQCH-AS1 | >0.3 | <0.05 |
| AC079466.1 | >0.3 | <0.05 |
| AL157838.1 | >0.3 | <0.05 |
| LINC01807 | >0.3 | <0.05 |
| AC080013.4 | >0.3 | <0.05 |
| AC023090.1 | >0.3 | <0.05 |
| ZNF32-AS1 | >0.3 | <0.05 |
| XIST | >0.3 | <0.05 |
| NUTM2A-AS1 | >0.3 | <0.05 |
| SNHG7 | >0.3 | <0.05 |
| LINC02298 | >0.3 | <0.05 |
| AP001033.1 | >0.3 | <0.05 |
| AC008735.4 | >0.3 | <0.05 |
| AL670729.1 | >0.3 | <0.05 |
| AP001107.9 | >0.3 | <0.05 |
| AL035587.1 | >0.3 | <0.05 |
| AC109347.2 | >0.3 | <0.05 |
| AC008556.1 | >0.3 | <0.05 |
| AC097639.1 | >0.3 | <0.05 |
| AL162586.1 | >0.3 | <0.05 |
| AC026401.3 | >0.3 | <0.05 |
| WASIR2 | >0.3 | <0.05 |
| LINC00648 | >0.3 | <0.05 |
| CASC22 | >0.3 | <0.05 |
| ZNF710-AS1 | >0.3 | <0.05 |
| AC010168.1 | >0.3 | <0.05 |
| AC087623.1 | >0.3 | <0.05 |
| HLA-DQB1-AS1 | >0.3 | <0.05 |
| AL132989.1 | >0.3 | <0.05 |
| RAB11B-AS1 | >0.3 | <0.05 |
| AC004264.1 | >0.3 | <0.05 |
| ST7-AS1 | >0.3 | <0.05 |
| LINC00958 | >0.3 | <0.05 |
| LINC02178 | >0.3 | <0.05 |
| AL121761.1 | >0.3 | <0.05 |
| AC022150.2 | >0.3 | <0.05 |
| AC010655.2 | >0.3 | <0.05 |
| NFYC-AS1 | >0.3 | <0.05 |
| AC040970.1 | >0.3 | <0.05 |
| NARF-IT1 | >0.3 | <0.05 |
| AL512598.1 | >0.3 | <0.05 |
| AL354696.1 | >0.3 | <0.05 |
| MHENCR | >0.3 | <0.05 |
| AC124248.1 | >0.3 | <0.05 |
| GAS5-AS1 | >0.3 | <0.05 |
| AL928654.2 | >0.3 | <0.05 |
| SERTAD4-AS1 | >0.3 | <0.05 |
| AC124016.2 | >0.3 | <0.05 |
| AL354993.2 | >0.3 | <0.05 |
| AP003352.1 | >0.3 | <0.05 |
| AC010618.2 | >0.3 | <0.05 |
| AC139795.2 | >0.3 | <0.05 |
| AC244197.2 | >0.3 | <0.05 |
| AC090517.2 | >0.3 | <0.05 |
| AC010329.1 | >0.3 | <0.05 |
| AC002401.4 | >0.3 | <0.05 |
| AC093620.1 | >0.3 | <0.05 |
| AC016727.1 | >0.3 | <0.05 |
| TRIM52-AS1 | >0.3 | <0.05 |
| AL445222.1 | >0.3 | <0.05 |
| AC009113.1 | >0.3 | <0.05 |
| HOTAIRM1 | >0.3 | <0.05 |
| AC108860.2 | >0.3 | <0.05 |
| AC020907.4 | >0.3 | <0.05 |
| THORLNC | >0.3 | <0.05 |
| AP001160.1 | >0.3 | <0.05 |
| AL137003.2 | >0.3 | <0.05 |
| AC092171.5 | >0.3 | <0.05 |
| PCAT19 | >0.3 | <0.05 |
| ERVH48-1 | >0.3 | <0.05 |
| LINC00355 | >0.3 | <0.05 |
| AC079414.3 | >0.3 | <0.05 |
| AC096586.2 | >0.3 | <0.05 |
| FLVCR1-DT | >0.3 | <0.05 |
| AL031716.1 | >0.3 | <0.05 |
| AL080317.1 | >0.3 | <0.05 |
| AL162431.2 | >0.3 | <0.05 |
| LINC01106 | >0.3 | <0.05 |
| ANKRD10-IT1 | >0.3 | <0.05 |
| MIR4435-2HG | >0.3 | <0.05 |
| LINC01871 | >0.3 | <0.05 |
| AC025259.3 | >0.3 | <0.05 |
| NEAT1 | >0.3 | <0.05 |
| AL021578.1 | >0.3 | <0.05 |
| AL031670.1 | >0.3 | <0.05 |
| AC079907.1 | >0.3 | <0.05 |
| PRKCZ-AS1 | >0.3 | <0.05 |
| LINC01705 | >0.3 | <0.05 |
| MIR205HG | >0.3 | <0.05 |
| AP001160.4 | >0.3 | <0.05 |
| AL118516.1 | >0.3 | <0.05 |
| BX284668.5 | >0.3 | <0.05 |
| AC010761.1 | >0.3 | <0.05 |
| AC011477.3 | >0.3 | <0.05 |
| AC090515.2 | >0.3 | <0.05 |
| PIK3IP1-AS1 | >0.3 | <0.05 |
| PSMB8-AS1 | >0.3 | <0.05 |
| AC004540.2 | >0.3 | <0.05 |
| AC090229.1 | >0.3 | <0.05 |
| LINC01011 | >0.3 | <0.05 |
| AC110285.2 | >0.3 | <0.05 |
| AL359881.1 | >0.3 | <0.05 |
| Z83851.1 | >0.3 | <0.05 |
| AC025165.4 | >0.3 | <0.05 |
| AC068338.3 | >0.3 | <0.05 |
| AC100860.1 | >0.3 | <0.05 |
| AP001469.3 | >0.3 | <0.05 |
| FAM111A-DT | >0.3 | <0.05 |
| AC016957.2 | >0.3 | <0.05 |
| AC009318.2 | >0.3 | <0.05 |
| AC093788.1 | >0.3 | <0.05 |
| AC009054.2 | >0.3 | <0.05 |
| AC005393.1 | >0.3 | <0.05 |
| LINC01612 | >0.3 | <0.05 |
| AC080112.1 | >0.3 | <0.05 |
| AC005041.3 | >0.3 | <0.05 |
| LINC00649 | >0.3 | <0.05 |
| AC104564.3 | >0.3 | <0.05 |
| NKILA | >0.3 | <0.05 |
| ZNF436-AS1 | >0.3 | <0.05 |
| AL133410.1 | >0.3 | <0.05 |
| AC019117.1 | >0.3 | <0.05 |
| PICSAR | >0.3 | <0.05 |
| GATA3-AS1 | >0.3 | <0.05 |
| AC132872.3 | >0.3 | <0.05 |
| LINC00709 | >0.3 | <0.05 |
| TP53TG1 | >0.3 | <0.05 |
| CASC15 | >0.3 | <0.05 |
| AC020558.2 | >0.3 | <0.05 |
| AC012360.3 | >0.3 | <0.05 |
| LINC00942 | >0.3 | <0.05 |
| FENDRR | >0.3 | <0.05 |
| AL049838.1 | >0.3 | <0.05 |
| AC106820.3 | >0.3 | <0.05 |
| AL450326.1 | >0.3 | <0.05 |
| AC067930.4 | >0.3 | <0.05 |
| AL158166.1 | >0.3 | <0.05 |
| BX322562.1 | >0.3 | <0.05 |
| AL033397.1 | >0.3 | <0.05 |
| SCARNA9 | >0.3 | <0.05 |
| AC005586.1 | >0.3 | <0.05 |
| AP001628.1 | >0.3 | <0.05 |
| AL135905.1 | >0.3 | <0.05 |
| AC108134.1 | >0.3 | <0.05 |
| TRHDE-AS1 | >0.3 | <0.05 |
| RNF157-AS1 | >0.3 | <0.05 |
| AC011468.5 | >0.3 | <0.05 |
| AC008915.2 | >0.3 | <0.05 |
| AC139100.2 | >0.3 | <0.05 |
| AC083880.1 | >0.3 | <0.05 |
| AC008610.1 | >0.3 | <0.05 |
| KMT2E-AS1 | >0.3 | <0.05 |
| AC011498.6 | >0.3 | <0.05 |
| FAM198B-AS1 | >0.3 | <0.05 |
| AC005746.3 | >0.3 | <0.05 |
| AC068888.1 | >0.3 | <0.05 |
| AL645940.1 | >0.3 | <0.05 |
| LINC01943 | >0.3 | <0.05 |
| CALML3-AS1 | >0.3 | <0.05 |
| AL359076.1 | >0.3 | <0.05 |
| AL645608.8 | >0.3 | <0.05 |
| GASAL1 | >0.3 | <0.05 |
| AC105277.1 | >0.3 | <0.05 |
| AL023284.4 | >0.3 | <0.05 |
| AP005899.1 | >0.3 | <0.05 |
| AC004223.3 | >0.3 | <0.05 |
| LINC02582 | >0.3 | <0.05 |
| AL122035.1 | >0.3 | <0.05 |
| LINC02154 | >0.3 | <0.05 |
| AL024508.2 | >0.3 | <0.05 |
| LINC01128 | >0.3 | <0.05 |
| AL591806.1 | >0.3 | <0.05 |
| GARS-DT | >0.3 | <0.05 |
| AL136295.7 | >0.3 | <0.05 |
| SDCBP2-AS1 | >0.3 | <0.05 |
| AC145423.2 | >0.3 | <0.05 |
| AC016590.3 | >0.3 | <0.05 |
| LINC02081 | >0.3 | <0.05 |
| AC092953.2 | >0.3 | <0.05 |
| AC018809.1 | >0.3 | <0.05 |
| AC004492.1 | >0.3 | <0.05 |
| OIP5-AS1 | >0.3 | <0.05 |
| AL023803.2 | >0.3 | <0.05 |
| AL591895.1 | >0.3 | <0.05 |
| AC078778.1 | >0.3 | <0.05 |
| AC137932.1 | >0.3 | <0.05 |
| AP003469.4 | >0.3 | <0.05 |
| AP001033.2 | >0.3 | <0.05 |
| LINC02315 | >0.3 | <0.05 |
| LINC01063 | >0.3 | <0.05 |
| AC073174.1 | >0.3 | <0.05 |
| LINC01977 | >0.3 | <0.05 |
| AC084357.2 | >0.3 | <0.05 |
| EIF3J-DT | >0.3 | <0.05 |
| FOXN3-AS1 | >0.3 | <0.05 |
| AC073389.1 | >0.3 | <0.05 |
| AC005332.4 | >0.3 | <0.05 |
| LINC00526 | >0.3 | <0.05 |
| PDXDC2P-NPIPB14P | >0.3 | <0.05 |
| AC127070.1 | >0.3 | <0.05 |
| AL136531.1 | >0.3 | <0.05 |
| PRKAG2-AS1 | >0.3 | <0.05 |
| AC004080.1 | >0.3 | <0.05 |
| AL133215.2 | >0.3 | <0.05 |
| AL355001.2 | >0.3 | <0.05 |
| AC104463.2 | >0.3 | <0.05 |
| AL139011.1 | >0.3 | <0.05 |
| AC095057.3 | >0.3 | <0.05 |
| AC099343.2 | >0.3 | <0.05 |
| TBX2-AS1 | >0.3 | <0.05 |
| NOP14-AS1 | >0.3 | <0.05 |
| HOXD-AS2 | >0.3 | <0.05 |
| AC005291.2 | >0.3 | <0.05 |
| AC245884.8 | >0.3 | <0.05 |
| AC104794.2 | >0.3 | <0.05 |
| N4BP2L2-IT2 | >0.3 | <0.05 |
| LINC01139 | >0.3 | <0.05 |
| LENG8-AS1 | >0.3 | <0.05 |
| LINC02012 | >0.3 | <0.05 |
| AP001160.3 | >0.3 | <0.05 |
| AC048344.4 | >0.3 | <0.05 |
| DM1-AS | >0.3 | <0.05 |
| AC133552.5 | >0.3 | <0.05 |
| AP000866.5 | >0.3 | <0.05 |
| LINC00641 | >0.3 | <0.05 |
| AC010487.2 | >0.3 | <0.05 |
| SMIM25 | >0.3 | <0.05 |
| KRT7-AS | >0.3 | <0.05 |
| AC002310.1 | >0.3 | <0.05 |
| GPRC5D-AS1 | >0.3 | <0.05 |
| GSEC | >0.3 | <0.05 |
| AL354793.1 | >0.3 | <0.05 |
| AC004812.2 | >0.3 | <0.05 |
| AC005332.3 | >0.3 | <0.05 |
| AL139260.1 | >0.3 | <0.05 |
| AC112491.1 | >0.3 | <0.05 |
| MBNL1-AS1 | >0.3 | <0.05 |
| AC100814.1 | >0.3 | <0.05 |
| AL391834.1 | >0.3 | <0.05 |
| MCM3AP-AS1 | >0.3 | <0.05 |
| AC100801.1 | >0.3 | <0.05 |
| AC004918.3 | >0.3 | <0.05 |
| AC036176.1 | >0.3 | <0.05 |
| AL591845.1 | >0.3 | <0.05 |
| AC138028.4 | >0.3 | <0.05 |
| AC079807.1 | >0.3 | <0.05 |
| AC102953.2 | >0.3 | <0.05 |
| AC087857.1 | >0.3 | <0.05 |
| AL121790.2 | >0.3 | <0.05 |
| PPP3CB-AS1 | >0.3 | <0.05 |
| BAIAP2-DT | >0.3 | <0.05 |
| AC003070.1 | >0.3 | <0.05 |
| AL118508.1 | >0.3 | <0.05 |
| AC024075.1 | >0.3 | <0.05 |
| AL645608.1 | >0.3 | <0.05 |
| AC092849.1 | >0.3 | <0.05 |
| AP000553.2 | >0.3 | <0.05 |
| AC005253.1 | >0.3 | <0.05 |
| AC004982.1 | >0.3 | <0.05 |
| AL357033.4 | >0.3 | <0.05 |
| AC104083.1 | >0.3 | <0.05 |
| SP2-AS1 | >0.3 | <0.05 |
| AC106900.2 | >0.3 | <0.05 |
| AL022328.1 | >0.3 | <0.05 |
| AC005332.6 | >0.3 | <0.05 |
| AC004471.1 | >0.3 | <0.05 |
| LINC00294 | >0.3 | <0.05 |
| AL353751.1 | >0.3 | <0.05 |
| ZSCAN16-AS1 | >0.3 | <0.05 |
| AC023509.4 | >0.3 | <0.05 |
| AC087752.3 | >0.3 | <0.05 |
| AC002553.2 | >0.3 | <0.05 |
| AL441992.1 | >0.3 | <0.05 |
| MIATNB | >0.3 | <0.05 |
| AC096992.2 | >0.3 | <0.05 |
| AC012510.1 | >0.3 | <0.05 |
| OCIAD1-AS1 | >0.3 | <0.05 |
| LINC01889 | >0.3 | <0.05 |
| AC018926.2 | >0.3 | <0.05 |
| AC099850.3 | >0.3 | <0.05 |
| AL121655.1 | >0.3 | <0.05 |
| CD27-AS1 | >0.3 | <0.05 |
| PTOV1-AS2 | >0.3 | <0.05 |
| AC099329.2 | >0.3 | <0.05 |
| CH17-340M24.3 | >0.3 | <0.05 |
| AL365356.5 | >0.3 | <0.05 |
| ARMCX5-GPRASP2 | >0.3 | <0.05 |
| AL117336.3 | >0.3 | <0.05 |
| STX17-AS1 | >0.3 | <0.05 |
| AC002398.1 | >0.3 | <0.05 |
| AC073389.3 | >0.3 | <0.05 |
| ARHGAP27P1-BPTFP1-KPNA2P3 | >0.3 | <0.05 |
| AL139123.1 | >0.3 | <0.05 |
| AL135999.1 | >0.3 | <0.05 |
| LINC01786 | >0.3 | <0.05 |
| AP001107.4 | >0.3 | <0.05 |
| AC008771.1 | >0.3 | <0.05 |
| DICER1-AS1 | >0.3 | <0.05 |
| AC084824.4 | >0.3 | <0.05 |
| PXN-AS1 | >0.3 | <0.05 |
| AC009005.1 | >0.3 | <0.05 |
| FLJ31356 | >0.3 | <0.05 |
| SPINT1-AS1 | >0.3 | <0.05 |
| AC005332.5 | >0.3 | <0.05 |
| AL365203.2 | >0.3 | <0.05 |
| LINC01116 | >0.3 | <0.05 |
| MIR181A2HG | >0.3 | <0.05 |
| LINC01089 | >0.3 | <0.05 |
| AL683813.1 | >0.3 | <0.05 |
| AC127024.6 | >0.3 | <0.05 |
| AC099518.2 | >0.3 | <0.05 |
| AC103702.2 | >0.3 | <0.05 |
| AC016888.1 | >0.3 | <0.05 |
| AC135050.5 | >0.3 | <0.05 |
| TMEM92-AS1 | >0.3 | <0.05 |
| AL033384.2 | >0.3 | <0.05 |
| AC091182.2 | >0.3 | <0.05 |
| AC137767.1 | >0.3 | <0.05 |
| HMGA1P4 | >0.3 | <0.05 |
| C5orf17 | >0.3 | <0.05 |
| AC009902.2 | >0.3 | <0.05 |
| AL359715.3 | >0.3 | <0.05 |
| LINC02035 | >0.3 | <0.05 |
| Z95115.1 | >0.3 | <0.05 |
| AC026471.2 | >0.3 | <0.05 |
| ARRDC1-AS1 | >0.3 | <0.05 |
| AL138781.1 | >0.3 | <0.05 |
| LINC01419 | >0.3 | <0.05 |
| AL049840.4 | >0.3 | <0.05 |
| AC114489.1 | >0.3 | <0.05 |
| LINC01521 | >0.3 | <0.05 |
| AC002553.1 | >0.3 | <0.05 |
| U62317.2 | >0.3 | <0.05 |
| AC011477.2 | >0.3 | <0.05 |
| LINC01833 | >0.3 | <0.05 |
| LINC00941 | >0.3 | <0.05 |
| AC062037.2 | >0.3 | <0.05 |
| NDUFB2-AS1 | >0.3 | <0.05 |
| AC005674.2 | >0.3 | <0.05 |
| LINC01748 | >0.3 | <0.05 |
| AL121895.2 | >0.3 | <0.05 |
| LINC00865 | >0.3 | <0.05 |
| AL121832.2 | >0.3 | <0.05 |
| AC037198.1 | >0.3 | <0.05 |
| AC007292.1 | >0.3 | <0.05 |
| AP001062.1 | >0.3 | <0.05 |
| CYTOR | >0.3 | <0.05 |
| AC024075.3 | >0.3 | <0.05 |
| AC120053.1 | >0.3 | <0.05 |
| AP002360.1 | >0.3 | <0.05 |
| AC012615.1 | >0.3 | <0.05 |
| AC005076.1 | >0.3 | <0.05 |
| AC006111.2 | >0.3 | <0.05 |
| MNX1-AS1 | >0.3 | <0.05 |
| AL049840.5 | >0.3 | <0.05 |
| AC005288.1 | >0.3 | <0.05 |
| AL139393.2 | >0.3 | <0.05 |
| AC023310.4 | >0.3 | <0.05 |
| AC015982.1 | >0.3 | <0.05 |
| PLBD1-AS1 | >0.3 | <0.05 |
| AC022150.4 | >0.3 | <0.05 |
| AC010761.3 | >0.3 | <0.05 |
| AL662844.4 | >0.3 | <0.05 |
| AL138724.1 | >0.3 | <0.05 |
| AL662791.1 | >0.3 | <0.05 |
| NDUFV2-AS1 | >0.3 | <0.05 |
| C1RL-AS1 | >0.3 | <0.05 |
| MUC20-OT1 | >0.3 | <0.05 |
| AC087741.1 | >0.3 | <0.05 |
| BX470102.1 | >0.3 | <0.05 |
| AC022509.2 | >0.3 | <0.05 |
| AC107464.3 | >0.3 | <0.05 |
| SNHG8 | >0.3 | <0.05 |
| BOLA3-AS1 | >0.3 | <0.05 |
| AL161752.1 | >0.3 | <0.05 |
| AC011450.1 | >0.3 | <0.05 |
| DANCR | >0.3 | <0.05 |
| AL158206.1 | >0.3 | <0.05 |
| AL596325.2 | >0.3 | <0.05 |
| AL390037.1 | >0.3 | <0.05 |
| AL050341.2 | >0.3 | <0.05 |
| AC005046.1 | >0.3 | <0.05 |
| TRPM2-AS | >0.3 | <0.05 |
| CAMTA1-DT | >0.3 | <0.05 |
| AC004253.1 | >0.3 | <0.05 |
| AL109811.3 | >0.3 | <0.05 |
| LINC01559 | >0.3 | <0.05 |
| HM13-IT1 | >0.3 | <0.05 |
| FBXL19-AS1 | >0.3 | <0.05 |
| AC010735.2 | >0.3 | <0.05 |
| MIR29B2CHG | >0.3 | <0.05 |
| LINC02585 | >0.3 | <0.05 |
| AC090739.1 | >0.3 | <0.05 |
| MINCR | >0.3 | <0.05 |
| AL390294.1 | >0.3 | <0.05 |
| AC253536.3 | >0.3 | <0.05 |
| AL583808.1 | >0.3 | <0.05 |
| AC138207.2 | >0.3 | <0.05 |
| AC008537.2 | >0.3 | <0.05 |
| AC005180.1 | >0.3 | <0.05 |
| AL356356.1 | >0.3 | <0.05 |
| AC124798.1 | >0.3 | <0.05 |
| SNHG17 | >0.3 | <0.05 |
| AL390729.1 | >0.3 | <0.05 |
| AL391244.3 | >0.3 | <0.05 |
| AC021028.1 | >0.3 | <0.05 |
| AL121839.2 | >0.3 | <0.05 |
| AC082651.3 | >0.3 | <0.05 |
| DLEU2 | >0.3 | <0.05 |
| CASC19 | >0.3 | <0.05 |
| AC073335.2 | >0.3 | <0.05 |
| AL139289.2 | >0.3 | <0.05 |
| LINC01213 | >0.3 | <0.05 |
| AL445472.1 | >0.3 | <0.05 |
| HOXA-AS2 | >0.3 | <0.05 |
| AC073534.1 | >0.3 | <0.05 |
| AC093726.1 | >0.3 | <0.05 |
| AC006435.2 | >0.3 | <0.05 |
| AC008760.1 | >0.3 | <0.05 |
| AC012645.4 | >0.3 | <0.05 |
| LINC02001 | >0.3 | <0.05 |
| FAM83C-AS1 | >0.3 | <0.05 |
| AC253576.2 | >0.3 | <0.05 |
| ELOA-AS1 | >0.3 | <0.05 |
| AC027575.2 | >0.3 | <0.05 |
| GAS6-DT | >0.3 | <0.05 |
| AC009506.1 | >0.3 | <0.05 |
| USP46-AS1 | >0.3 | <0.05 |
| AL022328.4 | >0.3 | <0.05 |
| LINC00174 | >0.3 | <0.05 |
| AC139887.1 | >0.3 | <0.05 |
| AL158847.1 | >0.3 | <0.05 |
| LINC00926 | >0.3 | <0.05 |
| AC091544.4 | >0.3 | <0.05 |
| LINC01534 | >0.3 | <0.05 |
| AC010463.3 | >0.3 | <0.05 |
| LINC00491 | >0.3 | <0.05 |
| AC025165.5 | >0.3 | <0.05 |
| AC105446.1 | >0.3 | <0.05 |
| AC068831.6 | >0.3 | <0.05 |
| AP000251.1 | >0.3 | <0.05 |
| AC017100.1 | >0.3 | <0.05 |
| PTOV1-AS1 | >0.3 | <0.05 |
| AC005387.1 | >0.3 | <0.05 |
| AC090114.2 | >0.3 | <0.05 |
| FLJ45513 | >0.3 | <0.05 |
| AC018521.6 | >0.3 | <0.05 |
| AF131216.3 | >0.3 | <0.05 |
| AC124067.2 | >0.3 | <0.05 |
| LINC01711 | >0.3 | <0.05 |
| AC022706.1 | >0.3 | <0.05 |
| AC034236.2 | >0.3 | <0.05 |
| DHRS4-AS1 | >0.3 | <0.05 |
| SNHG21 | >0.3 | <0.05 |
| TRAPPC12-AS1 | >0.3 | <0.05 |
| AC067852.2 | >0.3 | <0.05 |
| MIR200CHG | >0.3 | <0.05 |
| AL022322.1 | >0.3 | <0.05 |
| AC010761.4 | >0.3 | <0.05 |
| LINC00265 | >0.3 | <0.05 |
| LINC00987 | >0.3 | <0.05 |
| AC003102.1 | >0.3 | <0.05 |
| THUMPD3-AS1 | >0.3 | <0.05 |
| AC092171.2 | >0.3 | <0.05 |
| AC133644.2 | >0.3 | <0.05 |
| AC145343.1 | >0.3 | <0.05 |
| AC136475.2 | >0.3 | <0.05 |
| AC007128.1 | >0.3 | <0.05 |
| NIFK-AS1 | >0.3 | <0.05 |
| AL354733.3 | >0.3 | <0.05 |
| CCDC18-AS1 | >0.3 | <0.05 |
| AC036214.2 | >0.3 | <0.05 |
| UCA1 | >0.3 | <0.05 |
| H1FX-AS1 | >0.3 | <0.05 |
| PCAT7 | >0.3 | <0.05 |
| RHPN1-AS1 | >0.3 | <0.05 |
| MIRLET7BHG | >0.3 | <0.05 |
| AC068473.5 | >0.3 | <0.05 |
| AC026369.1 | >0.3 | <0.05 |
| AC007991.2 | >0.3 | <0.05 |
| AL512791.1 | >0.3 | <0.05 |
| CBR3-AS1 | >0.3 | <0.05 |
| LINC02542 | >0.3 | <0.05 |
| AL359921.1 | >0.3 | <0.05 |
| AC245595.1 | >0.3 | <0.05 |
| AP001453.2 | >0.3 | <0.05 |
| AC117498.2 | >0.3 | <0.05 |
| MANCR | >0.3 | <0.05 |
| AC068768.1 | >0.3 | <0.05 |
| AC114488.1 | >0.3 | <0.05 |
| AC004771.3 | >0.3 | <0.05 |
| AC073508.3 | >0.3 | <0.05 |
| AC016065.1 | >0.3 | <0.05 |
| SNHG18 | >0.3 | <0.05 |
| MYCNUT | >0.3 | <0.05 |
| AL136295.2 | >0.3 | <0.05 |
| LINC02432 | >0.3 | <0.05 |
| LINC02474 | >0.3 | <0.05 |
| EPB41L4A-DT | >0.3 | <0.05 |
| G2E3-AS1 | >0.3 | <0.05 |
| AP002807.1 | >0.3 | <0.05 |
| AC245041.2 | >0.3 | <0.05 |
| AC027373.1 | >0.3 | <0.05 |
| AL157394.1 | >0.3 | <0.05 |
| AC012186.2 | >0.3 | <0.05 |
| ADIRF-AS1 | >0.3 | <0.05 |
| AC096751.2 | >0.3 | <0.05 |
| AC004908.2 | >0.3 | <0.05 |
| LINC01023 | >0.3 | <0.05 |
| AP000786.1 | >0.3 | <0.05 |
| AC006538.1 | >0.3 | <0.05 |
| FARP1-AS1 | >0.3 | <0.05 |
| AL049555.1 | >0.3 | <0.05 |
| MIR4713HG | >0.3 | <0.05 |
| AL133338.1 | >0.3 | <0.05 |
| ST3GAL5-AS1 | >0.3 | <0.05 |
| AC008393.1 | >0.3 | <0.05 |
| AL157392.3 | >0.3 | <0.05 |
| AC025171.2 | >0.3 | <0.05 |
| SNHG16 | >0.3 | <0.05 |
| AC005746.2 | >0.3 | <0.05 |
| AC004597.1 | >0.3 | <0.05 |
| AC008105.1 | >0.3 | <0.05 |
| AC093297.2 | >0.3 | <0.05 |
| AC139530.1 | >0.3 | <0.05 |
| AC012313.1 | >0.3 | <0.05 |
| MALAT1 | >0.3 | <0.05 |
| SCAMP1-AS1 | >0.3 | <0.05 |
| LINC00847 | >0.3 | <0.05 |
| AL049840.1 | >0.3 | <0.05 |
| AC135050.3 | >0.3 | <0.05 |
| SNHG14 | >0.3 | <0.05 |
| AL031186.1 | >0.3 | <0.05 |
| Z84484.1 | >0.3 | <0.05 |
| AC106047.1 | >0.3 | <0.05 |
| AC093278.2 | >0.3 | <0.05 |
| AL391001.1 | >0.3 | <0.05 |
| ALDH1L1-AS2 | >0.3 | <0.05 |
| LINC02004 | >0.3 | <0.05 |
| AC010326.3 | >0.3 | <0.05 |
| AC135050.6 | >0.3 | <0.05 |
| LINC01679 | >0.3 | <0.05 |
| AC110792.3 | >0.3 | <0.05 |
| AC005005.3 | >0.3 | <0.05 |
| MCCC1-AS1 | >0.3 | <0.05 |
| AL136304.1 | >0.3 | <0.05 |
| DLG5-AS1 | >0.3 | <0.05 |
| NR2F1-AS1 | >0.3 | <0.05 |
| AL049629.1 | >0.3 | <0.05 |
| SOS1-IT1 | >0.3 | <0.05 |
| AL360181.1 | >0.3 | <0.05 |
| AC008014.1 | >0.3 | <0.05 |
| AC090948.3 | >0.3 | <0.05 |
| AL355353.1 | >0.3 | <0.05 |
| AL163051.2 | >0.3 | <0.05 |
| PITPNA-AS1 | >0.3 | <0.05 |
| BLACAT1 | >0.3 | <0.05 |
| AC113346.1 | >0.3 | <0.05 |
| AC104532.2 | >0.3 | <0.05 |
| AC009570.1 | >0.3 | <0.05 |
| AC092171.3 | >0.3 | <0.05 |
| AC005261.1 | >0.3 | <0.05 |
| AP003068.1 | >0.3 | <0.05 |
| TDRKH-AS1 | >0.3 | <0.05 |
| AC002044.1 | >0.3 | <0.05 |
| AC011442.1 | >0.3 | <0.05 |
| AC068152.1 | >0.3 | <0.05 |
| AL022328.2 | >0.3 | <0.05 |
| KCCAT333 | >0.3 | <0.05 |
| AC008759.2 | >0.3 | <0.05 |
| AC105460.1 | >0.3 | <0.05 |
| CAHM | >0.3 | <0.05 |
| AF117829.1 | >0.3 | <0.05 |
| LINC00342 | >0.3 | <0.05 |
| AL109615.3 | >0.3 | <0.05 |
| AL135818.2 | >0.3 | <0.05 |
| AC007038.2 | >0.3 | <0.05 |
| UBL7-AS1 | >0.3 | <0.05 |
| LINC01980 | >0.3 | <0.05 |
| AC092295.2 | >0.3 | <0.05 |
| AC233728.1 | >0.3 | <0.05 |
| TOB1-AS1 | >0.3 | <0.05 |
| AC022167.2 | >0.3 | <0.05 |
| AL021707.8 | >0.3 | <0.05 |
| AL139385.1 | >0.3 | <0.05 |
| LINC01410 | >0.3 | <0.05 |
| AL355574.1 | >0.3 | <0.05 |
| AC232271.1 | >0.3 | <0.05 |
| AC016737.1 | >0.3 | <0.05 |
| AC104758.2 | >0.3 | <0.05 |
| VIM-AS1 | >0.3 | <0.05 |
| AL606834.1 | >0.3 | <0.05 |
| ATP1B3-AS1 | >0.3 | <0.05 |
| AC007938.3 | >0.3 | <0.05 |
| AL162424.1 | >0.3 | <0.05 |
| AC022075.1 | >0.3 | <0.05 |
| LINC01311 | >0.3 | <0.05 |
| TSPEAR-AS2 | >0.3 | <0.05 |
| AL137793.1 | >0.3 | <0.05 |
| AC024075.2 | >0.3 | <0.05 |
| AL031673.1 | >0.3 | <0.05 |
| AC091729.3 | >0.3 | <0.05 |
| ACVR2B-AS1 | >0.3 | <0.05 |
| AP003486.1 | >0.3 | <0.05 |
| AL132712.1 | >0.3 | <0.05 |
| AC100861.1 | >0.3 | <0.05 |
| AC108134.3 | >0.3 | <0.05 |
| LINC00324 | >0.3 | <0.05 |
| AC074033.1 | >0.3 | <0.05 |
| AC010883.1 | >0.3 | <0.05 |
| CYP4F26P | >0.3 | <0.05 |
| AC009118.3 | >0.3 | <0.05 |
| AC007996.1 | >0.3 | <0.05 |
| PSORS1C3 | >0.3 | <0.05 |
| AC138696.2 | >0.3 | <0.05 |
| LINC01224 | >0.3 | <0.05 |
| AC006504.5 | >0.3 | <0.05 |
| AC091153.3 | >0.3 | <0.05 |
| SCHLAP1 | >0.3 | <0.05 |
| AC145098.1 | >0.3 | <0.05 |
| AL035661.1 | >0.3 | <0.05 |
| AL354919.2 | >0.3 | <0.05 |
| AL035071.1 | >0.3 | <0.05 |
| AC253536.6 | >0.3 | <0.05 |
| AC093484.4 | >0.3 | <0.05 |
| AC019080.5 | >0.3 | <0.05 |
| AL355075.2 | >0.3 | <0.05 |
| FOXD3-AS1 | >0.3 | <0.05 |
| AC007566.1 | >0.3 | <0.05 |
| FLNB-AS1 | >0.3 | <0.05 |
| AC027348.1 | >0.3 | <0.05 |
| STAG3L5P-PVRIG2P-PILRB | >0.3 | <0.05 |
| AL590652.1 | >0.3 | <0.05 |
| SNHG4 | >0.3 | <0.05 |
| AC027601.3 | >0.3 | <0.05 |
| AP004608.1 | >0.3 | <0.05 |
| AC093227.1 | >0.3 | <0.05 |
| AC097641.2 | >0.3 | <0.05 |
| SNHG20 | >0.3 | <0.05 |
| AC108134.4 | >0.3 | <0.05 |
| AC099568.2 | >0.3 | <0.05 |
| TTC28-AS1 | >0.3 | <0.05 |
| AC114730.3 | >0.3 | <0.05 |
| SLC25A25-AS1 | >0.3 | <0.05 |
| AL355607.1 | >0.3 | <0.05 |
| TMEM51-AS1 | >0.3 | <0.05 |
| AC073365.1 | >0.3 | <0.05 |
| AC145423.3 | >0.3 | <0.05 |
| AL161729.4 | >0.3 | <0.05 |
| ZNF790-AS1 | >0.3 | <0.05 |
| CRNDE | >0.3 | <0.05 |
| AC015849.3 | >0.3 | <0.05 |
| AC021016.2 | >0.3 | <0.05 |
| AC092535.4 | >0.3 | <0.05 |
| AC020891.2 | >0.3 | <0.05 |
| FGF14-AS2 | >0.3 | <0.05 |
| GAS5 | >0.3 | <0.05 |
| LINC01608 | >0.3 | <0.05 |
| AL359715.1 | >0.3 | <0.05 |
| AC009318.3 | >0.3 | <0.05 |
| AC083967.1 | >0.3 | <0.05 |
| AP002840.2 | >0.3 | <0.05 |
| AC104695.3 | >0.3 | <0.05 |
| EVX1-AS | >0.3 | <0.05 |
| LINC02595 | >0.3 | <0.05 |
| AC026356.1 | >0.3 | <0.05 |
| LINC00115 | >0.3 | <0.05 |
| AC006449.6 | >0.3 | <0.05 |
| HCG18 | >0.3 | <0.05 |
| AL117379.1 | >0.3 | <0.05 |
| AL645608.6 | >0.3 | <0.05 |
| ERVK13-1 | >0.3 | <0.05 |
| AC015813.1 | >0.3 | <0.05 |
| AC103706.1 | >0.3 | <0.05 |
| AC004069.1 | >0.3 | <0.05 |
| AL451165.2 | >0.3 | <0.05 |
| AL691482.3 | >0.3 | <0.05 |
| AL137003.1 | >0.3 | <0.05 |
| AL359921.2 | >0.3 | <0.05 |
| ACTA2-AS1 | >0.3 | <0.05 |
| AL121601.1 | >0.3 | <0.05 |
| MAFG-DT | >0.3 | <0.05 |
| AC073569.2 | >0.3 | <0.05 |
| AC005993.1 | >0.3 | <0.05 |
| AC008735.2 | >0.3 | <0.05 |
