# Supplementary material for: A hypoxia related long non-coding RNA signature could accurately predict survival outcomes in patients with bladder cancer
Source: Bioengineered. 2021 Jul 19;12(1):3802–23. doi: 10.1080/21655979.2021.1948781 (PMC8806425; doi:10.1080/21655979.2021.1948781)
Supplement: Supplemental Material [file KBIE_A_1948781_SM4898.zip › supplementary/Supplementary Table 3.docx]

Supplementary Table 3. Univariate Cox regression analysis

| gene | KM | B | SE | HR | HR.95L | HR.95H | pvalue |
| --- | --- | --- | --- | --- | --- | --- | --- |
| AC020663.2 | 0.005 | -0.402 | 0.176 | 0.669 | 0.474 | 0.945 | 0.022 |
| AL035563.1 | 0.001 | -0.417 | 0.158 | 0.659 | 0.483 | 0.899 | 0.009 |
| AC116914.2 | 0.003 | -0.353 | 0.099 | 0.703 | 0.579 | 0.853 | 0.000 |
| AP003419.3 | 0.043 | -0.377 | 0.127 | 0.686 | 0.535 | 0.880 | 0.003 |
| AC010503.4 | 0.001 | -0.027 | 0.008 | 0.974 | 0.958 | 0.990 | 0.001 |
| AL021154.1 | 0.035 | -0.380 | 0.154 | 0.684 | 0.506 | 0.924 | 0.013 |
| AC010331.1 | 0.000 | -0.372 | 0.124 | 0.689 | 0.540 | 0.880 | 0.003 |
| RAD51-AS1 | 0.028 | -0.125 | 0.056 | 0.882 | 0.791 | 0.985 | 0.025 |
| AC005726.3 | 0.000 | -0.126 | 0.049 | 0.881 | 0.800 | 0.971 | 0.011 |
| AF131215.5 | 0.023 | -0.200 | 0.101 | 0.818 | 0.672 | 0.997 | 0.047 |
| AL355488.1 | 0.038 | -0.123 | 0.054 | 0.885 | 0.796 | 0.983 | 0.022 |
| AL158212.2 | 0.009 | -0.369 | 0.177 | 0.692 | 0.489 | 0.979 | 0.037 |
| AC009065.4 | 0.007 | -0.246 | 0.101 | 0.782 | 0.642 | 0.952 | 0.015 |
| AC078880.3 | 0.015 | -0.220 | 0.085 | 0.802 | 0.679 | 0.947 | 0.009 |
| AL450384.2 | 0.000 | -0.178 | 0.053 | 0.837 | 0.754 | 0.929 | 0.001 |
| LINC01004 | 0.002 | -0.187 | 0.067 | 0.829 | 0.727 | 0.947 | 0.006 |
| AGAP2-AS1 | 0.033 | 0.029 | 0.013 | 1.030 | 1.004 | 1.056 | 0.023 |
| AL359091.5 | 0.006 | 0.072 | 0.036 | 1.075 | 1.002 | 1.153 | 0.045 |
| AC006042.1 | 0.006 | -0.068 | 0.031 | 0.935 | 0.879 | 0.994 | 0.031 |
| AC008074.2 | 0.000 | -0.540 | 0.186 | 0.583 | 0.404 | 0.840 | 0.004 |
| AC092171.4 | 0.003 | -0.222 | 0.113 | 0.801 | 0.642 | 0.999 | 0.049 |
| AC018638.7 | 0.007 | -0.570 | 0.163 | 0.565 | 0.411 | 0.778 | 0.000 |
| AC092794.1 | 0.001 | -0.554 | 0.271 | 0.575 | 0.338 | 0.978 | 0.041 |
| ZNF426-DT | 0.006 | -0.232 | 0.090 | 0.793 | 0.664 | 0.946 | 0.010 |
| TMEM161B-AS1 | 0.012 | -0.297 | 0.132 | 0.743 | 0.574 | 0.962 | 0.024 |
| AC004148.2 | 0.006 | -0.199 | 0.059 | 0.819 | 0.730 | 0.920 | 0.001 |
| AC012615.6 | 0.003 | -0.560 | 0.213 | 0.571 | 0.376 | 0.867 | 0.009 |
| AC080129.2 | 0.002 | -0.397 | 0.153 | 0.672 | 0.498 | 0.907 | 0.009 |
| AC008124.1 | 0.040 | -0.321 | 0.115 | 0.726 | 0.579 | 0.910 | 0.005 |
| AL136084.3 | 0.002 | 0.266 | 0.078 | 1.304 | 1.120 | 1.519 | 0.001 |
| AC005840.4 | 0.001 | -0.408 | 0.114 | 0.665 | 0.531 | 0.831 | 0.000 |
| AC063948.1 | 0.010 | -0.279 | 0.124 | 0.756 | 0.593 | 0.964 | 0.024 |
| AC015912.3 | 0.043 | -0.039 | 0.018 | 0.962 | 0.928 | 0.996 | 0.031 |
| ZNF460-AS1 | 0.000 | -0.660 | 0.200 | 0.517 | 0.349 | 0.765 | 0.001 |
| IPO5P1 | 0.004 | -0.080 | 0.024 | 0.924 | 0.880 | 0.969 | 0.001 |
| RBPMS-AS1 | 0.015 | -0.247 | 0.116 | 0.781 | 0.623 | 0.980 | 0.033 |
| AL138756.1 | 0.007 | -0.303 | 0.127 | 0.739 | 0.576 | 0.948 | 0.017 |
| PPP1R26-AS1 | 0.041 | -0.602 | 0.204 | 0.547 | 0.367 | 0.817 | 0.003 |
| AL049840.3 | 0.026 | 0.204 | 0.083 | 1.227 | 1.043 | 1.443 | 0.014 |
| AL513329.1 | 0.018 | -0.408 | 0.160 | 0.665 | 0.486 | 0.910 | 0.011 |
| AC104785.1 | 0.000 | -0.492 | 0.185 | 0.611 | 0.425 | 0.879 | 0.008 |
| AL513320.1 | 0.031 | -0.331 | 0.137 | 0.718 | 0.549 | 0.939 | 0.016 |
| ZNF213-AS1 | 0.003 | -0.177 | 0.071 | 0.838 | 0.729 | 0.962 | 0.012 |
| AL139349.1 | 0.001 | -0.244 | 0.108 | 0.783 | 0.634 | 0.968 | 0.024 |
| LINC02195 | 0.049 | -0.083 | 0.041 | 0.920 | 0.849 | 0.998 | 0.044 |
| AC009065.8 | 0.000 | -0.274 | 0.095 | 0.760 | 0.631 | 0.917 | 0.004 |
| ZKSCAN2-DT | 0.013 | -0.422 | 0.148 | 0.656 | 0.491 | 0.876 | 0.004 |
| AL031714.1 | 0.045 | -0.326 | 0.135 | 0.722 | 0.554 | 0.940 | 0.016 |
| AC006942.1 | 0.029 | -0.279 | 0.138 | 0.756 | 0.578 | 0.990 | 0.042 |
| AC018904.1 | 0.008 | -0.023 | 0.012 | 0.977 | 0.955 | 1.000 | 0.046 |
| AC062017.1 | 0.014 | -0.481 | 0.151 | 0.618 | 0.460 | 0.831 | 0.001 |
| AL390728.6 | 0.000 | -0.076 | 0.020 | 0.927 | 0.891 | 0.965 | 0.000 |
| PSMA3-AS1 | 0.017 | -0.128 | 0.046 | 0.880 | 0.805 | 0.962 | 0.005 |
| AL353708.3 | 0.028 | -0.453 | 0.200 | 0.635 | 0.430 | 0.940 | 0.023 |
| AC132807.2 | 0.047 | -0.089 | 0.045 | 0.915 | 0.837 | 0.999 | 0.048 |
| AC105942.1 | 0.024 | 0.072 | 0.020 | 1.074 | 1.033 | 1.117 | 0.000 |
| AC005009.1 | 0.030 | -0.373 | 0.141 | 0.689 | 0.523 | 0.907 | 0.008 |
| AL031775.1 | 0.000 | -0.444 | 0.103 | 0.642 | 0.525 | 0.784 | 0.000 |
| LINC01560 | 0.049 | -0.160 | 0.075 | 0.852 | 0.736 | 0.987 | 0.033 |
| AC116667.1 | 0.007 | -0.411 | 0.165 | 0.663 | 0.480 | 0.916 | 0.013 |
| MSC-AS1 | 0.031 | 0.117 | 0.049 | 1.124 | 1.021 | 1.237 | 0.017 |
| AL513218.1 | 0.040 | -0.481 | 0.151 | 0.618 | 0.459 | 0.831 | 0.001 |
| AC068620.2 | 0.031 | -0.643 | 0.224 | 0.526 | 0.339 | 0.815 | 0.004 |
| AC108673.3 | 0.005 | -0.130 | 0.057 | 0.878 | 0.786 | 0.981 | 0.021 |
| AC026801.2 | 0.048 | -0.192 | 0.083 | 0.825 | 0.701 | 0.970 | 0.020 |
| LINC01767 | 0.000 | -0.203 | 0.082 | 0.816 | 0.695 | 0.958 | 0.013 |
| AL031775.2 | 0.003 | -0.382 | 0.129 | 0.682 | 0.530 | 0.878 | 0.003 |
| LINC01355 | 0.004 | -0.212 | 0.080 | 0.809 | 0.691 | 0.946 | 0.008 |
| AC046143.2 | 0.001 | -0.142 | 0.066 | 0.867 | 0.762 | 0.988 | 0.032 |
| AL513477.2 | 0.016 | -0.487 | 0.176 | 0.615 | 0.436 | 0.867 | 0.006 |
| AC083862.2 | 0.013 | -0.492 | 0.181 | 0.611 | 0.429 | 0.872 | 0.007 |
| AL139289.1 | 0.045 | -0.334 | 0.149 | 0.716 | 0.534 | 0.960 | 0.025 |
| AC104825.1 | 0.001 | -0.157 | 0.047 | 0.854 | 0.780 | 0.936 | 0.001 |
| AL121944.1 | 0.005 | -0.181 | 0.070 | 0.834 | 0.727 | 0.957 | 0.010 |
| AC020910.4 | 0.006 | -0.169 | 0.058 | 0.845 | 0.754 | 0.947 | 0.004 |
| LINC002481 | 0.010 | -0.309 | 0.099 | 0.734 | 0.604 | 0.892 | 0.002 |
| AC073896.4 | 0.020 | -0.031 | 0.014 | 0.970 | 0.943 | 0.997 | 0.028 |
| AL390719.2 | 0.009 | -0.025 | 0.012 | 0.976 | 0.954 | 0.998 | 0.035 |
| AL139089.1 | 0.005 | -0.368 | 0.117 | 0.692 | 0.551 | 0.870 | 0.002 |
| AC090198.1 | 0.006 | -0.422 | 0.171 | 0.656 | 0.469 | 0.917 | 0.014 |
| TNFRSF14-AS1 | 0.000 | -0.359 | 0.102 | 0.698 | 0.572 | 0.852 | 0.000 |
| LINC02544 | 0.050 | 0.041 | 0.015 | 1.042 | 1.011 | 1.073 | 0.007 |
| AL358472.3 | 0.005 | -0.234 | 0.108 | 0.791 | 0.640 | 0.978 | 0.030 |
| GEMIN7-AS1 | 0.004 | -0.449 | 0.178 | 0.638 | 0.450 | 0.905 | 0.012 |
| AC005261.3 | 0.001 | -0.120 | 0.041 | 0.887 | 0.819 | 0.960 | 0.003 |
| ERVE-1 | 0.009 | -0.059 | 0.029 | 0.943 | 0.890 | 0.999 | 0.046 |
| THAP9-AS1 | 0.027 | -0.068 | 0.026 | 0.934 | 0.888 | 0.983 | 0.008 |
| AC011468.1 | 0.004 | -0.252 | 0.066 | 0.777 | 0.682 | 0.885 | 0.000 |
| AC104695.2 | 0.001 | -0.110 | 0.045 | 0.895 | 0.820 | 0.978 | 0.014 |
| AC090948.1 | 0.006 | -0.557 | 0.241 | 0.573 | 0.357 | 0.918 | 0.021 |
| AC093726.2 | 0.003 | -0.529 | 0.198 | 0.589 | 0.400 | 0.868 | 0.007 |
| AC018695.6 | 0.009 | -0.072 | 0.030 | 0.930 | 0.878 | 0.986 | 0.015 |
| AC010542.5 | 0.003 | -0.168 | 0.054 | 0.846 | 0.761 | 0.940 | 0.002 |
| AP002026.1 | 0.008 | -0.180 | 0.072 | 0.835 | 0.725 | 0.962 | 0.013 |
| AC020765.2 | 0.001 | -0.173 | 0.073 | 0.841 | 0.730 | 0.971 | 0.018 |
| MIR193BHG | 0.035 | 0.072 | 0.030 | 1.075 | 1.014 | 1.140 | 0.016 |
| LINC00930 | 0.001 | -0.066 | 0.023 | 0.936 | 0.894 | 0.980 | 0.005 |
| AC008035.1 | 0.016 | -0.225 | 0.092 | 0.798 | 0.666 | 0.956 | 0.014 |
| AL021707.3 | 0.027 | -0.316 | 0.129 | 0.729 | 0.566 | 0.939 | 0.014 |
| PAXIP1-AS1 | 0.003 | -0.062 | 0.028 | 0.940 | 0.891 | 0.993 | 0.026 |
| USP30-AS1 | 0.037 | -0.137 | 0.044 | 0.872 | 0.799 | 0.951 | 0.002 |
| AC009690.2 | 0.004 | -0.588 | 0.267 | 0.555 | 0.329 | 0.938 | 0.028 |
| AC009812.1 | 0.026 | -0.228 | 0.086 | 0.796 | 0.672 | 0.943 | 0.008 |
| AC002128.1 | 0.002 | -0.523 | 0.179 | 0.593 | 0.417 | 0.842 | 0.004 |
| AC074117.1 | 0.010 | -0.214 | 0.072 | 0.807 | 0.701 | 0.930 | 0.003 |
| AL354836.1 | 0.028 | -0.028 | 0.012 | 0.972 | 0.949 | 0.996 | 0.025 |
| CFAP58-DT | 0.048 | 0.092 | 0.029 | 1.096 | 1.035 | 1.160 | 0.002 |
| AC027020.2 | 0.005 | -0.498 | 0.149 | 0.608 | 0.454 | 0.813 | 0.001 |
| AL162258.2 | 0.000 | -0.595 | 0.186 | 0.552 | 0.383 | 0.795 | 0.001 |
| AL731567.1 | 0.013 | -0.085 | 0.030 | 0.918 | 0.866 | 0.973 | 0.004 |
| AC008687.3 | 0.044 | -0.261 | 0.128 | 0.771 | 0.600 | 0.990 | 0.042 |
| AL008582.1 | 0.035 | -0.585 | 0.209 | 0.557 | 0.370 | 0.840 | 0.005 |
| C5orf56 | 0.010 | -0.399 | 0.169 | 0.671 | 0.482 | 0.935 | 0.018 |
| SH3BP5-AS1 | 0.000 | -0.159 | 0.069 | 0.853 | 0.745 | 0.977 | 0.022 |
| AC024060.1 | 0.004 | -0.113 | 0.028 | 0.893 | 0.845 | 0.944 | 0.000 |
| AC018653.3 | 0.007 | -0.274 | 0.101 | 0.760 | 0.624 | 0.926 | 0.006 |
| LINC00893 | 0.011 | -0.330 | 0.168 | 0.719 | 0.518 | 0.999 | 0.049 |
| AC092119.2 | 0.016 | -0.438 | 0.194 | 0.645 | 0.441 | 0.943 | 0.024 |
| TNRC6C-AS1 | 0.007 | -0.094 | 0.043 | 0.910 | 0.836 | 0.990 | 0.029 |
| AC007998.4 | 0.004 | -0.083 | 0.037 | 0.920 | 0.855 | 0.990 | 0.026 |
| AC010168.2 | 0.000 | -0.407 | 0.125 | 0.666 | 0.521 | 0.851 | 0.001 |
| AC009120.2 | 0.000 | -0.340 | 0.105 | 0.712 | 0.579 | 0.874 | 0.001 |
| AL162586.1 | 0.001 | -0.390 | 0.114 | 0.677 | 0.542 | 0.845 | 0.001 |
| WASIR2 | 0.000 | -0.208 | 0.100 | 0.812 | 0.668 | 0.988 | 0.037 |
| AC022150.2 | 0.001 | -0.183 | 0.058 | 0.833 | 0.744 | 0.933 | 0.002 |
| AL354696.1 | 0.037 | -0.368 | 0.153 | 0.692 | 0.512 | 0.935 | 0.016 |
| AP003352.1 | 0.000 | -0.162 | 0.049 | 0.850 | 0.772 | 0.936 | 0.001 |
| AL445222.1 | 0.001 | -0.230 | 0.096 | 0.795 | 0.658 | 0.960 | 0.017 |
| HOTAIRM1 | 0.048 | -0.018 | 0.008 | 0.983 | 0.968 | 0.997 | 0.022 |
| AC092171.5 | 0.050 | -0.069 | 0.033 | 0.933 | 0.874 | 0.997 | 0.039 |
| LINC01106 | 0.006 | -0.443 | 0.179 | 0.642 | 0.453 | 0.912 | 0.013 |
| LINC01871 | 0.043 | -0.054 | 0.021 | 0.947 | 0.909 | 0.987 | 0.011 |
| AL118516.1 | 0.029 | -0.056 | 0.025 | 0.946 | 0.900 | 0.994 | 0.028 |
| AC011477.3 | 0.037 | -0.120 | 0.049 | 0.887 | 0.806 | 0.976 | 0.014 |
| AC090515.2 | 0.042 | -0.307 | 0.139 | 0.736 | 0.560 | 0.967 | 0.028 |
| PSMB8-AS1 | 0.045 | -0.068 | 0.021 | 0.935 | 0.897 | 0.974 | 0.001 |
| AC090229.1 | 0.029 | -0.233 | 0.108 | 0.792 | 0.641 | 0.980 | 0.032 |
| LINC01011 | 0.001 | -0.587 | 0.188 | 0.556 | 0.385 | 0.804 | 0.002 |
| AC025165.4 | 0.001 | -0.508 | 0.223 | 0.602 | 0.389 | 0.931 | 0.023 |
| AC068338.3 | 0.013 | -0.232 | 0.108 | 0.793 | 0.641 | 0.981 | 0.032 |
| FAM111A-DT | 0.014 | -0.284 | 0.093 | 0.753 | 0.627 | 0.903 | 0.002 |
| AC016957.2 | 0.006 | -0.700 | 0.188 | 0.496 | 0.344 | 0.717 | 0.000 |
| AC093788.1 | 0.000 | -0.498 | 0.147 | 0.608 | 0.456 | 0.811 | 0.001 |
| AC080112.1 | 0.038 | -0.043 | 0.015 | 0.958 | 0.931 | 0.986 | 0.004 |
| LINC00649 | 0.001 | -0.372 | 0.138 | 0.689 | 0.526 | 0.904 | 0.007 |
| AC104564.3 | 0.000 | -0.605 | 0.179 | 0.546 | 0.385 | 0.775 | 0.001 |
| ZNF436-AS1 | 0.002 | -0.153 | 0.058 | 0.858 | 0.765 | 0.962 | 0.008 |
| AL133410.1 | 0.000 | -0.542 | 0.156 | 0.581 | 0.428 | 0.790 | 0.001 |
| GATA3-AS1 | 0.003 | -0.032 | 0.013 | 0.968 | 0.944 | 0.993 | 0.014 |
| AC132872.3 | 0.004 | -0.244 | 0.091 | 0.784 | 0.656 | 0.936 | 0.007 |
| LINC00709 | 0.047 | -0.301 | 0.131 | 0.740 | 0.573 | 0.956 | 0.021 |
| TP53TG1 | 0.009 | -0.010 | 0.005 | 0.990 | 0.981 | 0.999 | 0.034 |
| LINC00942 | 0.013 | 0.017 | 0.006 | 1.017 | 1.005 | 1.028 | 0.004 |
| BX322562.1 | 0.038 | 0.182 | 0.080 | 1.199 | 1.025 | 1.403 | 0.023 |
| AC008610.1 | 0.004 | -0.172 | 0.062 | 0.842 | 0.746 | 0.950 | 0.005 |
| AC005746.3 | 0.013 | -0.153 | 0.065 | 0.858 | 0.755 | 0.975 | 0.019 |
| AL645940.1 | 0.004 | -0.409 | 0.175 | 0.664 | 0.472 | 0.935 | 0.019 |
| AL024508.2 | 0.049 | -0.234 | 0.078 | 0.791 | 0.679 | 0.922 | 0.003 |
| GARS-DT | 0.042 | -0.392 | 0.191 | 0.676 | 0.465 | 0.982 | 0.040 |
| AC018809.1 | 0.049 | -0.475 | 0.155 | 0.622 | 0.459 | 0.843 | 0.002 |
| AL591895.1 | 0.050 | -0.024 | 0.010 | 0.977 | 0.958 | 0.996 | 0.016 |
| AC078778.1 | 0.014 | -0.430 | 0.114 | 0.650 | 0.520 | 0.813 | 0.000 |
| PDXDC2P-NPIPB14P | 0.001 | -0.197 | 0.086 | 0.822 | 0.694 | 0.972 | 0.022 |
| AC099343.2 | 0.000 | -0.642 | 0.190 | 0.526 | 0.363 | 0.763 | 0.001 |
| AC245884.8 | 0.001 | -0.354 | 0.121 | 0.702 | 0.554 | 0.889 | 0.003 |
| LENG8-AS1 | 0.022 | -0.213 | 0.075 | 0.809 | 0.698 | 0.936 | 0.004 |
| KRT7-AS | 0.000 | -0.024 | 0.008 | 0.976 | 0.960 | 0.993 | 0.005 |
| AC005332.3 | 0.002 | -0.074 | 0.034 | 0.929 | 0.869 | 0.993 | 0.030 |
| AC100814.1 | 0.033 | -0.221 | 0.083 | 0.802 | 0.681 | 0.944 | 0.008 |
| AL121790.2 | 0.019 | -0.090 | 0.038 | 0.914 | 0.849 | 0.985 | 0.018 |
| AL357033.4 | 0.002 | -0.205 | 0.084 | 0.814 | 0.691 | 0.960 | 0.014 |
| OCIAD1-AS1 | 0.003 | -0.490 | 0.150 | 0.612 | 0.457 | 0.821 | 0.001 |
| AC018926.2 | 0.048 | -0.483 | 0.176 | 0.617 | 0.437 | 0.871 | 0.006 |
| AC099850.3 | 0.031 | 0.028 | 0.011 | 1.028 | 1.007 | 1.050 | 0.009 |
| PTOV1-AS2 | 0.000 | -0.136 | 0.038 | 0.873 | 0.810 | 0.941 | 0.000 |
| ARHGAP27P1  -BPTFP1-KPNA2P3 | 0.036 | -0.125 | 0.051 | 0.883 | 0.798 | 0.976 | 0.015 |
| AL139123.1 | 0.022 | -0.421 | 0.194 | 0.656 | 0.449 | 0.960 | 0.030 |
| AL135999.1 | 0.046 | -0.290 | 0.142 | 0.748 | 0.566 | 0.988 | 0.041 |
| AC099518.2 | 0.000 | -0.495 | 0.132 | 0.610 | 0.471 | 0.790 | 0.000 |
| AL033384.2 | 0.007 | -0.347 | 0.121 | 0.707 | 0.557 | 0.897 | 0.004 |
| HMGA1P4 | 0.034 | -0.125 | 0.056 | 0.883 | 0.791 | 0.985 | 0.026 |
| AC011477.2 | 0.004 | -0.153 | 0.054 | 0.858 | 0.771 | 0.954 | 0.005 |
| LINC01833 | 0.017 | -0.113 | 0.043 | 0.893 | 0.821 | 0.972 | 0.009 |
| AC005674.2 | 0.010 | -0.550 | 0.179 | 0.577 | 0.406 | 0.819 | 0.002 |
| AL121895.2 | 0.029 | -0.258 | 0.103 | 0.772 | 0.631 | 0.945 | 0.012 |
| AC007292.1 | 0.005 | -0.230 | 0.109 | 0.795 | 0.642 | 0.983 | 0.034 |
| AC120053.1 | 0.001 | -0.192 | 0.056 | 0.826 | 0.740 | 0.922 | 0.001 |
| AC012615.1 | 0.019 | -0.128 | 0.056 | 0.880 | 0.789 | 0.982 | 0.022 |
| AL662844.4 | 0.024 | -0.867 | 0.266 | 0.420 | 0.250 | 0.708 | 0.001 |
| C1RL-AS1 | 0.012 | -0.428 | 0.169 | 0.652 | 0.468 | 0.908 | 0.011 |
| AC087741.1 | 0.006 | -0.267 | 0.083 | 0.765 | 0.651 | 0.900 | 0.001 |
| TRPM2-AS | 0.014 | -0.071 | 0.035 | 0.932 | 0.870 | 0.998 | 0.044 |
| AC004253.1 | 0.026 | -0.344 | 0.138 | 0.709 | 0.541 | 0.929 | 0.013 |
| AL390294.1 | 0.000 | -0.080 | 0.032 | 0.923 | 0.868 | 0.982 | 0.012 |
| AC073335.2 | 0.000 | -0.100 | 0.034 | 0.905 | 0.846 | 0.968 | 0.004 |
| AC073534.1 | 0.015 | -0.611 | 0.185 | 0.543 | 0.377 | 0.780 | 0.001 |
| AC093726.1 | 0.009 | -0.204 | 0.075 | 0.815 | 0.703 | 0.945 | 0.007 |
| AC008760.1 | 0.006 | -0.210 | 0.082 | 0.810 | 0.690 | 0.951 | 0.010 |
| AC253576.2 | 0.001 | -0.285 | 0.135 | 0.752 | 0.577 | 0.979 | 0.035 |
| AL022328.4 | 0.039 | -0.343 | 0.145 | 0.710 | 0.534 | 0.942 | 0.018 |
| AC091544.4 | 0.001 | -0.120 | 0.054 | 0.887 | 0.799 | 0.985 | 0.025 |
| AC034236.2 | 0.000 | -0.587 | 0.162 | 0.556 | 0.405 | 0.764 | 0.000 |
| MIR200CHG | 0.017 | -0.012 | 0.005 | 0.988 | 0.978 | 0.998 | 0.014 |
| AL022322.1 | 0.000 | -0.219 | 0.069 | 0.804 | 0.702 | 0.920 | 0.002 |
| AC010761.4 | 0.005 | -0.400 | 0.163 | 0.670 | 0.487 | 0.922 | 0.014 |
| THUMPD3-AS1 | 0.002 | -0.165 | 0.054 | 0.848 | 0.762 | 0.943 | 0.002 |
| SNHG18 | 0.028 | -0.011 | 0.004 | 0.989 | 0.981 | 0.998 | 0.015 |
| AL136295.2 | 0.001 | -0.743 | 0.221 | 0.476 | 0.309 | 0.734 | 0.001 |
| AP002807.1 | 0.016 | -0.231 | 0.074 | 0.794 | 0.686 | 0.919 | 0.002 |
| AC093297.2 | 0.048 | -0.147 | 0.054 | 0.863 | 0.776 | 0.960 | 0.007 |
| SCAMP1-AS1 | 0.046 | -0.213 | 0.063 | 0.808 | 0.714 | 0.914 | 0.001 |
| AC135050.3 | 0.028 | -0.474 | 0.193 | 0.622 | 0.427 | 0.908 | 0.014 |
| Z84484.1 | 0.000 | -0.719 | 0.215 | 0.487 | 0.320 | 0.742 | 0.001 |
| AC010326.3 | 0.007 | -0.086 | 0.027 | 0.918 | 0.871 | 0.968 | 0.002 |
| LINC01679 | 0.017 | -0.222 | 0.109 | 0.801 | 0.647 | 0.992 | 0.042 |
| NR2F1-AS1 | 0.029 | 0.428 | 0.122 | 1.533 | 1.208 | 1.947 | 0.000 |
| AC090948.3 | 0.008 | -0.431 | 0.170 | 0.650 | 0.466 | 0.907 | 0.011 |
| AL355353.1 | 0.008 | -0.054 | 0.019 | 0.948 | 0.912 | 0.984 | 0.005 |
| AC104532.2 | 0.004 | -0.510 | 0.165 | 0.600 | 0.434 | 0.830 | 0.002 |
| AC005261.1 | 0.001 | -0.104 | 0.032 | 0.901 | 0.847 | 0.960 | 0.001 |
| AL022328.2 | 0.009 | -0.199 | 0.069 | 0.819 | 0.715 | 0.938 | 0.004 |
| AC008759.2 | 0.019 | -0.281 | 0.104 | 0.755 | 0.616 | 0.926 | 0.007 |
| AC007038.2 | 0.001 | -0.246 | 0.096 | 0.782 | 0.647 | 0.944 | 0.011 |
| AL021707.8 | 0.000 | -0.181 | 0.054 | 0.835 | 0.751 | 0.928 | 0.001 |
| AL139385.1 | 0.019 | 0.331 | 0.111 | 1.392 | 1.119 | 1.731 | 0.003 |
| AC232271.1 | 0.018 | -0.361 | 0.173 | 0.697 | 0.497 | 0.978 | 0.037 |
| AC016737.1 | 0.030 | -0.629 | 0.227 | 0.533 | 0.341 | 0.832 | 0.006 |
| AC022075.1 | 0.000 | -0.067 | 0.027 | 0.935 | 0.887 | 0.985 | 0.011 |
| LINC01311 | 0.001 | -0.280 | 0.130 | 0.756 | 0.585 | 0.976 | 0.032 |
| LINC00324 | 0.018 | -0.215 | 0.083 | 0.806 | 0.685 | 0.949 | 0.009 |
| CYP4F26P | 0.016 | -0.225 | 0.110 | 0.798 | 0.644 | 0.990 | 0.040 |
| PSORS1C3 | 0.007 | -0.038 | 0.017 | 0.963 | 0.932 | 0.995 | 0.022 |
| AL354919.2 | 0.030 | -0.125 | 0.039 | 0.882 | 0.817 | 0.953 | 0.002 |
| AC027348.1 | 0.022 | -0.203 | 0.093 | 0.816 | 0.681 | 0.979 | 0.029 |
| STAG3L5P  -PVRIG2P-PILRB | 0.000 | -0.664 | 0.172 | 0.515 | 0.368 | 0.721 | 0.000 |
| AL590652.1 | 0.046 | -0.213 | 0.105 | 0.808 | 0.658 | 0.994 | 0.043 |
| AC093227.1 | 0.020 | -0.320 | 0.103 | 0.726 | 0.594 | 0.889 | 0.002 |
| SNHG20 | 0.036 | -0.185 | 0.072 | 0.831 | 0.721 | 0.957 | 0.010 |
| TTC28-AS1 | 0.005 | -0.531 | 0.160 | 0.588 | 0.430 | 0.804 | 0.001 |
| AC114730.3 | 0.027 | -0.328 | 0.159 | 0.720 | 0.527 | 0.984 | 0.039 |
| SLC25A25-AS1 | 0.001 | -0.280 | 0.084 | 0.756 | 0.641 | 0.892 | 0.001 |
| TMEM51-AS1 | 0.036 | -0.258 | 0.081 | 0.773 | 0.660 | 0.905 | 0.001 |
| AL359715.1 | 0.040 | -0.177 | 0.080 | 0.838 | 0.716 | 0.980 | 0.027 |
| LINC00115 | 0.009 | -0.546 | 0.198 | 0.579 | 0.393 | 0.854 | 0.006 |
| AC103706.1 | 0.014 | -0.109 | 0.044 | 0.897 | 0.822 | 0.978 | 0.014 |
| AL359921.2 | 0.036 | -0.151 | 0.064 | 0.860 | 0.759 | 0.975 | 0.018 |
| MAFG-DT | 0.003 | 0.060 | 0.017 | 1.062 | 1.028 | 1.097 | 0.000 |
| AC008735.2 | 0.023 | -0.082 | 0.031 | 0.922 | 0.867 | 0.980 | 0.009 |
