# Supplementary material for: A hypoxia related long non-coding RNA signature could accurately predict survival outcomes in patients with bladder cancer
Source: Bioengineered. 2021 Jul 19;12(1):3802–23. doi: 10.1080/21655979.2021.1948781 (PMC8806425; doi:10.1080/21655979.2021.1948781)
Supplement: Supplemental Material [file KBIE_A_1948781_SM4898.zip › supplementary/Supplementary Table 4.docx]

Supplementary Table 4. lncRNA-mRNA links among lncRNAs and mRNAs

| mRNA | lncRNA | cor | corPval |
| --- | --- | --- | --- |
| ISG20 | USP30-AS1 | 0.739197121 | 9.93E-73 |
| ISG20 | PSMB8-AS1 | 0.498971884 | 1.90E-27 |
| GALK1 | MAFG-DT | 0.441667759 | 3.40E-21 |
| B3GALT6 | MAFG-DT | 0.441592575 | 3.46E-21 |
| ANKZF1 | STAG3L5P-PVRIG2P-PILRB | 0.421643329 | 2.83E-19 |
| ANXA2 | AC024060.1 | -0.41199141 | 2.15E-18 |
| KLHL24 | AL162586.1 | 0.411766339 | 2.25E-18 |
| CXCR4 | PSMB8-AS1 | 0.400526049 | 2.20E-17 |
| SLC2A5 | PSMB8-AS1 | 0.391387341 | 1.32E-16 |
| P4HA2 | AC024060.1 | -0.37560934 | 2.55E-15 |
| ANKZF1 | AC024060.1 | 0.375127904 | 2.79E-15 |
| EXT1 | AC024060.1 | -0.36866147 | 8.96E-15 |
| RRAGD | AC105942.1 | 0.368430648 | 9.33E-15 |
| HSPA5 | AP003352.1 | -0.36325904 | 2.33E-14 |
| HDLBP | AC024060.1 | -0.36017364 | 3.98E-14 |
| MYH9 | AC024060.1 | -0.3580154 | 5.78E-14 |
| ENO3 | MAFG-DT | 0.354748181 | 1.01E-13 |
| SLC2A5 | USP30-AS1 | 0.35196008 | 1.62E-13 |
| NR3C1 | AC024060.1 | -0.35056894 | 2.04E-13 |
| PGM1 | AP003352.1 | -0.34942376 | 2.48E-13 |
| NFIL3 | AC024060.1 | -0.3492425 | 2.55E-13 |
| PGM1 | AC024060.1 | -0.34707126 | 3.66E-13 |
| LDHA | AC024060.1 | -0.34524276 | 4.94E-13 |
| PGK1 | AC024060.1 | -0.3451612 | 5.01E-13 |
| TGFB3 | AC105942.1 | 0.345153944 | 5.02E-13 |
| HSPA5 | AC024060.1 | -0.34256934 | 7.66E-13 |
| NDST1 | AP003352.1 | -0.34250885 | 7.73E-13 |
| FBP1 | AC024060.1 | 0.34149927 | 9.11E-13 |
| MT2A | USP30-AS1 | 0.341429912 | 9.21E-13 |
| CXCR4 | USP30-AS1 | 0.339583866 | 1.24E-12 |
| ZNF292 | AL162586.1 | 0.336078488 | 2.17E-12 |
| PLAC8 | USP30-AS1 | 0.33519016 | 2.50E-12 |
| FAM162A | AC024060.1 | 0.333315978 | 3.37E-12 |
| PLAUR | USP30-AS1 | 0.333032007 | 3.52E-12 |
| TGM2 | USP30-AS1 | 0.329907469 | 5.74E-12 |
| HDLBP | AP003352.1 | -0.32980386 | 5.83E-12 |
| NAGK | AC024060.1 | 0.32879083 | 6.82E-12 |
| NAGK | AL031775.1 | 0.328746916 | 6.87E-12 |
| CAVIN1 | PSMB8-AS1 | 0.328299105 | 7.36E-12 |
| ANXA2 | AP003352.1 | -0.32616097 | 1.02E-11 |
| UGP2 | AP003352.1 | -0.32550628 | 1.13E-11 |
| WSB1 | STAG3L5P-PVRIG2P-PILRB | 0.325028234 | 1.22E-11 |
| ENO1 | AC024060.1 | -0.32274694 | 1.72E-11 |
| MT2A | PSMB8-AS1 | 0.322059877 | 1.91E-11 |
| BGN | AC105942.1 | 0.321436204 | 2.10E-11 |
| PGK1 | AP003352.1 | -0.32137093 | 2.12E-11 |
| P4HA2 | AP003352.1 | -0.32059462 | 2.38E-11 |
| NDST1 | AC024060.1 | -0.31259843 | 7.77E-11 |
| MIF | AP003352.1 | 0.312472761 | 7.92E-11 |
| FAM162A | AP003352.1 | 0.310124691 | 1.11E-10 |
| P4HA2 | AC016957.2 | -0.30959137 | 1.20E-10 |
| HSPA5 | AC016957.2 | -0.30818989 | 1.47E-10 |
| PGK1 | AC016957.2 | -0.30792507 | 1.53E-10 |
| WSB1 | AL162586.1 | 0.306812704 | 1.79E-10 |
| CAVIN1 | AP003352.1 | -0.30635943 | 1.91E-10 |
| DCN | AC105942.1 | 0.305794735 | 2.07E-10 |
| NR3C1 | AP003352.1 | -0.30493396 | 2.34E-10 |
| TPBG | AP003352.1 | -0.30379171 | 2.75E-10 |
| SDC3 | AC105942.1 | 0.301834605 | 3.62E-10 |
| MYH9 | AP003352.1 | -0.30122287 | 3.94E-10 |
| SLC25A1 | AP003352.1 | 0.300100502 | 4.61E-10 |
| WSB1 | AC024060.1 | 0.300066939 | 4.63E-10 |
